# The search for yield predictors for mature field-grown plants from juvenile pot-grown cassava (*Manihot esculenta* Crantz)

**DOI:** 10.1371/journal.pone.0232595

**Published:** 2020-05-06

**Authors:** Michael O. Adu, Paul A. Asare, David O. Yawson, Mishael A. Nyarko, Ahmed Abdul Razak, Amoah K. Kusi, Josiah W. Tachie-Menson, Emmanuel Afutu, Dick A. Andoh, Frank K. Ackah, Grace C. Vanderpuije, Kingsley J. Taah, Elvis Asare-Bediako, Godwin Amenorpe

**Affiliations:** 1 Department of Crop Science, School of Agriculture, College of Agriculture and Natural Sciences, University of Cape Coast, Cape Coast, Ghana; 2 Centre for Resource Management and Environmental Studies (CERMES), The University of the West Indies, Bridgetown, Barbados; 3 Nuclear Agricultural Research, Biotechnology and Nuclear Agriculture Research Institute, Ghana Atomic Energy Commission, Legon, Accra, Ghana; University of Helsinki, FINLAND

## Abstract

Cassava is the 6^th^ most important source of dietary energy in the world but its root system architecture (RSA) had seldom been quantified. Ability to select superior genotypes at juvenile stages can significantly reduce the cost and time for breeding to bridge the large yield gap. This study adopted a simple approach to phenotyping RSA traits of juvenile and mature cassava plants to identify genotypic differences and the relationships between juvenile traits and harvest index of mature plants. Root classes were categorised and root and shoot traits of eight (8) juvenile pot-grown cassava genotypes, were measured at 30 and 45 days after planting (DAP). The same or related traits were measured at 7 months after planting of the same genotypes grown in the field while yield and yield components were measured in 12-months old field-grown plants. The field experiment was done in 2017 and repeated in 2018. Differences between genotypes for the measured traits were explored using analysis of variance (ANOVA) while traits in juvenile plants were correlated or regressed onto traits measured in 7- and 12-months old plants. The results show significant genotypic variations for most of the traits measured in both juvenile and 7-months old plants. In the 12-months old plants, differences between genotypes were consistent for both 2017 and 2018. Broad-sense heritability was highest for the number of commercial roots (0.87) and shoot fresh weight (0.78) and intermediate for the total number of roots (0.60), harvest index (0.58), fresh weight of roots (0.45). For all the sampling time points or growth stages, there were greater correlations between traits measured at a particular growth stage than between the same traits at different growth stages. However, some juvenile-mature plant trait relationships were significant, positive and consistent for both 2017 and 2018. For example, total root length and the total number of roots in 30 DAP, and branching density of upper nodal roots in 45 DAP, positively correlated with harvest index of 12-months old plants in both 2017 and 2018. Similarly, the diameter of nodal roots, for example, had a negative, significant correlation with fresh shoot biomass of mature plants in both 2017 and 2018. Regression of traits measured in 30 DAP explained up to 22% and 36% of the variation in HI of mature plants in 2017 and 2018, respectively. It is concluded that the simple, rapid, inexpensive phenotyping approach adopted in this study is robust for identifying genotypic variations in juvenile cassava using root system traits. Also, the results provide seminal evidence for the existence of useful relationships between traits of juvenile and mature cassava plants that can be explored to predict yield and yield components.

## 1.0 Introduction

Cassava (*Manihot esculenta* Crantz) is the sixth major source of dietary energy in the world, with over 70% of global production used for human consumption and the remaining serving industrial, feed use, and other purposes [1]. According to the FAOSTAT Database, global production in 2017 was approximately 292 million tonnes from an area of 26.3 million ha. In terms of quantity produced, cassava is the most important root crop in Ghana, covering over 22% of the total area of land grown to food crops, with per capita consumption of about 153 kg/year [2]. Cassava also contributes to Ghana’s export earnings and has accounted for about 16–46% of the agricultural GDP over time [3, 4]. Increases in production in Ghana and Sub-Saharan Africa (SSA) have been driven mainly by expansion in area harvested rather than yield gains [5]. Current yields in SSA are about 8-fold below potential yields [6]. Yield in Ghana is between 5.0 and 11.8t/ha although the potential yield is estimated around 30t/ha [7]. Poor agronomic management in some environments is most likely a major contributory factor to the large reported yield gap. In SSA, for instance, cassava is largely produced on marginal soils and or under low external input conditions due to its resilience. However, the proportion of yield gap relevant to plant breeding might be the difference between potential yield in each environment, combined with the best cultivar and agronomic practice [6, 8].

The potential for improving soil resource acquisition and use efficiency on poor soils, typical of many regions of SSA, through improvement in root system architecture (RSA) of crop plants is now widely recognized [9–11]. The ability to balance trade-offs between marketable roots and traits that drive nutrient and water uptake would be key to improving the productivity of cassava even in resource-poor environments [12]. Yet, being a root crop, only a few studies have attempted to quantify cassava RSA, let alone establish relationships between root traits of juvenile cassava plants and harvest index of mature plants. Previous studies on cassava roots have mainly concentrated on bulking and qualities of the storage root [13]. Studies on root system development including root growth, root branching, dry matter distribution and geometric root traits such as root area and width have been conducted [12, 14–20]. Thus, there are limited quantitative studies on cassava RSA and genotypic variation in non-storage root traits. Some studies have focused on root length and the development and/or quantification of adventitious roots emerging from cutting without tuber bulking and lateral roots on the adventitious roots [13, 16]. Few studies have focused on the branching pattern of cassava roots even though this might be critical in root distribution and soil exploration for the acquisition of nutrients and other resources [16, 21, 22].

Difficulty in rapidly and accurately phenotyping cassava root traits are some of the bottlenecks that need to be removed for RSA traits to be quantified and used as input for improving yields of the crop. In addition to the general difficulties associated with root system studies of any crop, other exclusive reasons have also constrained quantitative root system studies of cassava. Cassava roots, the main economic part of the crop, could be as long as 2m deep in the ground [16], making excavation and quantitative analysis cumbersome, prone to inaccuracies, and ill-suited to screening large genetic populations. The ability to screen for variation in cassava root traits in the early stages of growth could circumvent some of the challenges and limitations outlined earlier and help identify useful root traits in genotypes. Traits related to shoot biomass, root diameter and branching density have the potential for use as predictors of water and nutrient use efficiency in crops [14] and might be useful for the selection of cassava genotypes during the early growth stages. The objectives of the current study were to measure the (i) genotypic variation in root traits of both juvenile and mature cassava genotypes; (ii) explore relationships between the yield of mature field-grown cassava and juvenile root traits.

## 2.0 Materials and methods

### 2.1 Plant material

The eight cassava genotypes used in this study have been previously described in [14]. One of the genotypes is released variety called ‘Capevars bankye’ (herein designated as ‘8H’). Five of the genotypes (designated 1A, 2B, 5E, 6F and 7G) have recently been recommended for release following field inspection by the National Variety Release and Registration Committee of the Ministry of Food and Agriculture (MoFA), Ghana. Genotypes 1A, 2B and 5E are yellow-fleshed varieties with high β-carotene and are suitable for local dishes and processing. Genotypes 6F and 7E are resistant to CMD and are suitable for local dishes and processing. The remaining genotypes included in the study are designated 3C and 4D. All the genotypes have similar time to tuber initiation and mature in 12 months.

### 2.2 Soil and environmental conditions

This study involved both field and pot experiments conducted at the Teaching and Research Farm at the University of Cape Coast (5° 06 N, 1° 15’ W). The study site experiences two seasons of rainfall with a peak in May to June and the minor in October. The dry season occurs between November and February [23]. The average precipitation recorded were 438 and 885 mm for the 2017 and 2018 cropping periods, respectively. The average temperatures were 25.8 and 23.9°C and the average relative humidity was 84.4 and 85.4% for the 2017 and 2018 cropping periods, respectively. Normal day length at the experimental site ranges from approximately 11.30 to 12.40 h [24], while solar radiation ranged from 17–30 MJ m^-2^ day^−1^ (mean ca 23 to 25 MJ m^-2^ day^−1^) day^−1^. The properties of the soil for both the field and pot experiments have been described previously [23].

### 2.3 Pot experiment

Topsoil (0–15 cm) was collected from a land very close to the plots for the field experiments. The setup in [14] was adopted for the pot experiment. In brief, nursery polybags of 45 cm long and 30 cm wide, with drainage holes at the bottom, were used as pots. The pots were filled with air-dried soil to a bulk density of approximately 1.1 g cm^-3^ and kept under a rain shelter. Before planting, the soil in each pot was watered with tap water to 80% field capacity (FC), determined gravimetrically, and allowed to drain overnight. Stem cuttings of approximately 20 cm of each genotype were randomly planted in the soil at an incline of about 45° from the centre of the pots, making sure that at least six nodes were within the soil. The cuttings were obtained from disease-free stems. Each genotype had eight replicates for each sampling period. Pots were positioned side by side with the assumption that pots’ spacing would not cause mutual shading of the plants at 45 days after planting (DAP). Positions of pots were rotated under the rain shelter every 10 days to reduce the effects of possible environmental gradients [14]. The soils in the pots were maintained at approximately 70% FC during the growth period by watering to weight with tap water every three to four days.

Harvesting or sampling was done at 30 and 45 days after planting (DAP). While we have previously shown that genetic variations in cassava root systems could be evident at 30 and 45 DAP [14], we also considered that screening at these early growth stages was appropriate based on our hypothesis that improving aspects of the juvenile root systems of cassava can accelerate access to soil resources, leading to rapid crop establishment and, consequently, greater yields. At each sampling, a blade was used to carefully cut through the longitudinal stitch lines on the sides of each pot (polybag) to expose the soil and the roots. The exposed soil and roots were then soaked in a basin of water for 3–4 minutes. Roots were removed and gently washed free of residual soil, using water hose at low pressure to minimize damages to roots [14]. Excavation and cleaning of roots were conducted by two individuals and they required an average of 6 minutes to complete the washing of one root system. Visual measurements of the root system were then conducted.

### 2.4 Field experiment

Two independent trials were conducted in 2017 and 2018. Maize and cowpea were normally grown at the site but plots for the experiments had been lying fallow for one and two years, respectively, for the 2017 and 2018 trials. Each of the trials was arranged in a randomized complete block design with two blocks containing the replications of each genotype. In both trials, each plot consisted of five rows, each measuring 10m long, with individual plants separated from one another by 1m in all directions and surrounded by a guard row. Stem cuttings were obtained and planted similarly as in the pot experiment. The experimental field had been slashed, ploughed and harrowed to a depth of about 30 cm, and was not ridged. The cuttings were directly planted into holes dug with a cutlass, typical of cassava planting in the region. In both 2017 and 2018, planting was done in May, typical of the main cassava planting season in Ghana. The plants were grown under rain-fed conditions and managed using best practices typical of the region where no external inputs are applied and weeds are controlled manually when necessary.

Twelve replicate plants within the middle section of the plots were used for analysis in both years. In each year, these plants were harvested 12 months after planting (12 MAP) for final yield parameters. At maturity, plants were excavated by a team of three or four people, using cutlasses, hoes and fork to loosen the soil, taking care not to damage any roots. Once the soil had been loosened, the roots were harvested by pulling the stem along with the roots out of the ground. In most cases, harvesting was done after rainfall to facilitate the process. In the few instances when roots were broken, they were also collected, added to the wholly excavated roots and taken to the laboratory for counting and measuring. In addition to the yield measurements in both years, in 2018, there was additional detailed shoot and root system analysis at 7 MAP, using 4 replicate plants per genotype per plot. In order not to disrupt planting density and the potential effect on yield at 12 MAP, each plot was arbitrarily divided into two in the 2018 trial. Plants from one-half of the plot were harvested at 7 MAP, leaving the plants in the other half of the plot to grow to maturity for the yield measurements at 12 MAP. Roots were excavated similarly as described previously.

### 2.5 Shoot and root system measurements

#### 2.5.1 Traits measured in pot-grown plants

**Fig 1:**
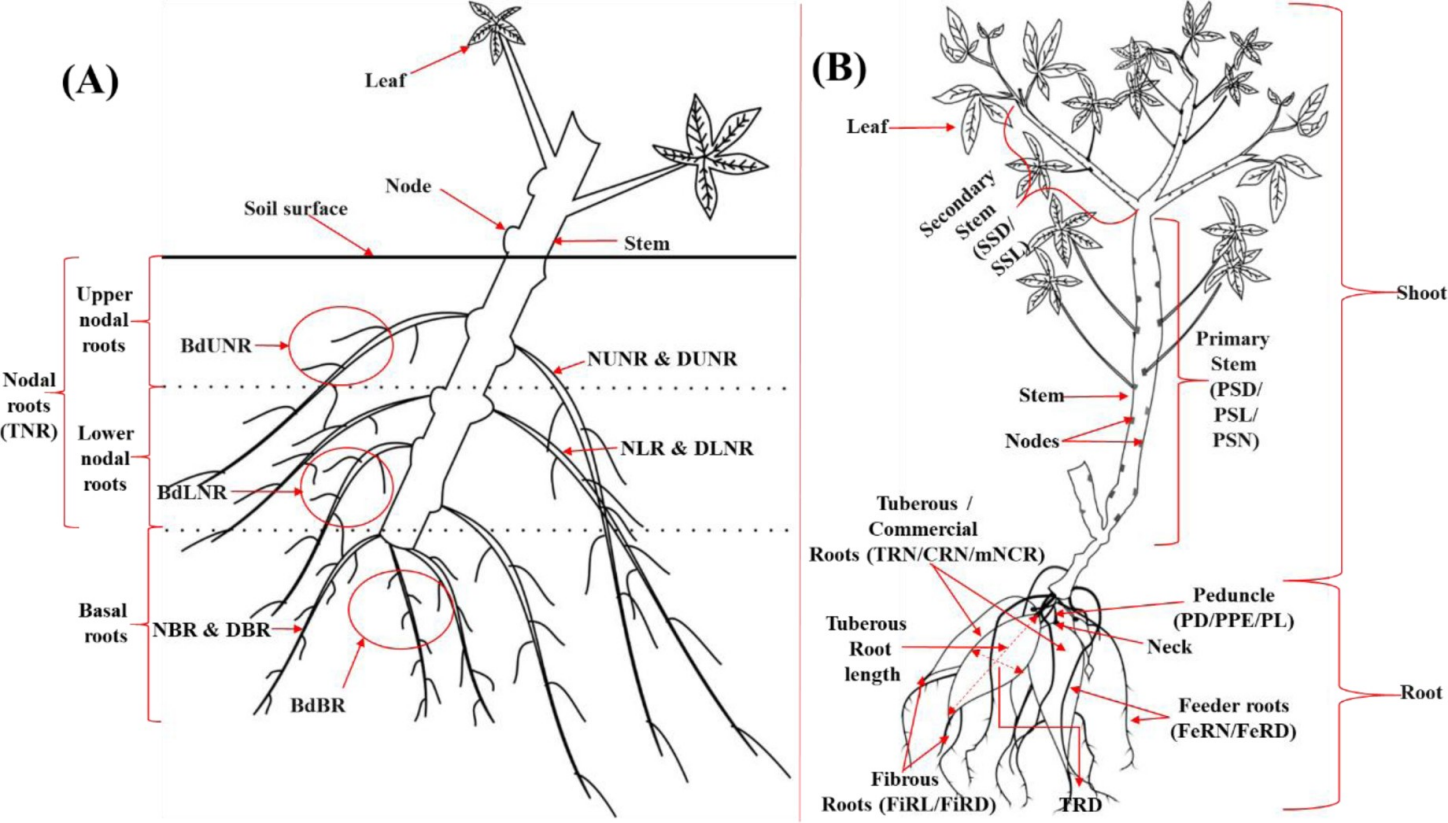
The categories given to pot-grown juvenile cassava plant **(A)** and field-grown 7-month old plant parts **(B)**. **A**: Upper nodal roots: emerged from the topmost nodes within the top 7 cm below the soil surface; lower nodal roots: emerged from the nodes on the stem cutting with 7–13 cm below the soil surface; basal roots: emerged from the callus at the base of the stem cutting. NUNR: number of upper nodal roots; DUNR: diameter of upper nodal roots; BdUNR: branching density of upper nodal roots; NLNR: number of the lower nodal root; DLNR: diameter of lower nodal roots, BdLNR: branching density of lower nodal roots; TNR: total number of nodal roots; NBR: number of basal roots; DBR: diameter of basal roots; BdBR: branching density of basal roots. **B**: The tuberous roots are the indeterminate, vegetative and starchy storage root that results from the swelling of primary root crown root; the commercial or marketable roots are the premium tuberous roots with no defects; the fibrous roots are the non-storage extensions of the tuberous roots and feeder roots here designate the small, non-storage roots that may be crucial in water and nutrients acquisition from the soil. SSD: secondary stem diameter; SSL: secondary stem length; PSD: primary stem diameter; PSL: primary stem length; PSN: primary stem number; TRD: tuberous roots diameter; TRL: tuberous roots length: TRN: tuberous roots number; FeRD: feeder roots diameter; FeRL: feeder roots length; FeRN: feeder roots number; FiRL: fibrous roots length; FiRD: fibrous roots diameter; PD: peduncle diameter; PE: peduncle extent; PL: peduncle length.

Summary of all measurements in this study is shown in Table 1. In the pot-grown plants, shoots, including the leaves, were weighed to determine shoot fresh weight (SFW) and oven-dried at 80°C for three days to determine shoot dry weight (SDW). Following root measurements, roots were weighed and oven-dried at 80°C for three days to determine root fresh weight (RFW) and root dry weight (RDW), respectively. The root-to-shoot ratio was calculated as the quotient of RDW and SDW. The protocol described by [14] was followed for the extraction and classification of root system features into three categories: upper nodal roots (UNRs), lower nodal roots (LNRs), and basal roots (BRs) (Fig 1A and Table 1). Total root length (TRL), the number of upper and lower nodal roots (NUNR and NLNR, respectively), the diameter of upper and lower nodal roots (DUNR and DLNR, respectively), and branching densities of upper and lower nodal roots (BdUNR and BdLNR, respectively), and the total number of nodal roots (TNR) were manually measured. Similarly, the number, diameter and branching density of basal roots (NBR, DBR, and BdBR, respectively) were measured. The root-to-shoot ratio was then estimated.

**Table 1:** Summary table of measurements with lists of all parameters measured, their units, acronyms and the time after planting and year the measurements were taken.

Stage and place of plant growth:		Pot-grown juvenile plants		Field-grown mature plants			
Period of measurement after planting:		30 DAP	45 DAP	7 MAP		12 MAP	
Parameters measured (units)	Acronym	Year of measurement					
		2017	2017	2017	2018	2017	2018
Shoot fresh weight (mg/kg)	SFW/ fSFW	✓	✓	-	✓	✓	✓
Shoot dry weight (mg)	SDW	✓	✓	-	-		
Root fresh weight (mg/kg)	RFW/ fRFW	✓	✓	-	✓	✓	✓
Root dry weight (mg)	RDW	✓	✓	-	-	-	-
Root-to-shoot ratio	R.S	✓	✓	-	-	-	-
Total root length (cm)	TRL	✓	✓	-	-	-	-
Number of upper nodal roots	NUNR	✓	✓	-	-	-	-
Diameter of upper nodal roots (mm)	DUNR	✓	✓	-	-	-	-
Branching density of upper nodal roots (roots cm^-1^)	BdUNR	✓	✓	-	-	-	-
Number of lower nodal roots	NLNR	✓	✓	-	-	-	-
Diameter of lower nodal roots (mm)	DLNR	✓	✓	-	-	-	-
Branching density of lower nodal roots (roots cm^-1^)	BdLNR	✓	✓	-	-	-	-
Total number of nodal roots	TNR	✓	✓	-	-	-	-
Number of basal roots	NBR	✓	✓	-	-	-	-
Diameter of basal roots (mm)	DBR	✓	✓	-	-	-	-
Branching density of basal roots (roots cm^-1^)	BdBR	✓	✓	-	-	-	-
Specific root length (mg cm^-1^)	SRL						
Branch level number	BN	-	-	-	✓	-	-
Commercial roots number	CRN	-	-	-	✓	✓	✓
Feeder roots diameter (mm)	FeRD	-	-	-	✓	-	-
Feeder roots length (cm)	FeRL	-	-	-	✓	-	-
Feeder roots number	FeRN	-	-	-	✓	-	-
Fibrous roots diameter (mm)	FiRD	-	-	-	✓	-	-
Fibrous roots length (cm)	FiRL	-	-	-	✓	-	-
Harvest index	HI	-	-	-	✓	✓	✓
Leafless stem height (cm)	LSH	-	-	-	✓	-	-
Peduncle diameter (mm)	PD	-	-	-	✓	-	-
Peduncle extent	PE	-	-	-	✓	-	-
Peduncle length (cm)	PL	-	-	-	✓	-	-
Primary stem diameter (mm)	PSD	-	-	-	✓	-	-
Primary stem length (cm)	PSL	-	-	-	✓	-	-
Primary stem number	PSN	-	-	-	✓	-	-
Secondary stem diameter (mm)	SSD	-	-	-	✓	-	-
Secondary stem length (cm)	SSL	-	-	-	✓	-	-
Tuberous roots diameter (mm)	TRD	-	-	-	✓	-	-
Tuberous roots length (cm)	fTRL	-	-	-	✓	-	-
Tuberous roots number	TRN	-	-	-	✓	-	-

Where three different acronyms are provided for a parameter, the acronyms are for measurements taken on 30 or 45 DAP, 7 MAP and 12 MAP, respectively. Checkmark (✓): parameter measured and dash (-): parameter not measured.

Three representative roots were randomly selected from each of the three root categories (Fig 1A) per plant for the measurement of root diameter and branching density. Root diameter for each root type was determined at three (3) cm from the stem cutting using digital callipers. Branching density for each category of the root was determined within six (6) cm distance. Total root length was measured by spreading and suspending the roots in water in a rectangular glass dish with a black background, taking care to avoid roots overlying on each other. Images of the total root system were captured with a Canon EOS 70D DSLR camera (https://www.usa.canon.com/) held stationary on a tripod at 50 cm above roots. Images were converted to a binary image and TRLs were extracted from root images using skeletonization routines [14] in ImageJ (US National Institutes of Health, Bethesda, MD, USA, https://imagej.nih.gov/ij/). Depending on the experience of the investigator, measurements on each juvenile root system took between 6 and 8 minutes.

#### 2.5.2 Traits measured at 7 MAP

Twenty-two traits were measured at 7 MAP and included branch level number (BN), commercial roots number (CRN), feeder roots diameter (FeRD), feeder roots length (FeRL), feeder roots number (FeRN) and fibrous roots diameter (FiRD) (Fig 1B). Other traits measured were fibrous roots length (FiRL), root fresh weight (fRFW), shoot fresh weight (fSFW), harvest index (HI), leafless stem height (LSH) and peduncle diameter (PD). Additionally, peduncle extent (PE), peduncle length (PL), primary stem diameter (PSD), primary stem length (PSL), primary stem number (PSN), secondary stem diameter (SSD), secondary stem length (SSL), tuberous roots diameter (TRD), tuberous roots length (TRL) and tuberous roots number (TRN) were measured at 7 months. BN, CRN, FeRN, PSN and TRN were counts of the number of divisions of vegetative branches, commercial or marketable roots, feeder (non-storage) roots, primary stems and tuberous (storage) roots, respectively. FeRD was measured with a calliper 3 cm from the insertion junction on main roots. Subsequently, the feeder roots were severed from the root system and their lengths measured with a ruler to obtain FeRL. The TRL was measured from the neck of the storage root to the beginning of the fibrous root at the end of the storage root. TRD was measured in the middle of the storage root. Fibrous roots are non-storage roots at the end of root tubers (Fig 1B). FiRD was measured in the middle of the fibrous root with a calliper and FiRL was measured with a ruler. LSH was measured with a tape measure from the soil surface to the longest branch next to the leaf-bearing branch. PSL was measured as the vertical height of the longest stem using a tape measure and from the soil surface to the first primary branch on the stem and the PSD was measured with a calliper in the middle of the primary stem. SSL was determined as the length of the first branch and the SSD determined with a calliper in the middle of the secondary stem. The most frequently occurring root peduncle extent (PE) was scored with a three-score system; 0 for sessile, 3 for pedunculated and 5 for mixed. Where applicable, PL was determined with a ruler and PE with a calliper in the middle of the peduncle. Fresh shoot and root biomass (fSFW and fRFW) were determined with measuring scale and used also to calculate HI.

#### 2.5.3 Traits measured in 12 MAP plants

At maturity, the above-ground material including stems and leaves were bulked together as shoot biomass and weighed. The yield parameters evaluated at maturity were the total number of roots, root fresh weight at maturity, the number of commercial roots at maturity, shoot fresh weight at maturity and root fresh weight at maturity, all calculated on a plant basis. Harvest index at maturity was calculated as the quotient of root fresh weight at maturity and total fresh biomass.

### 2.6 Statistical analyses

Analyses for the 30 and 45 DAP pot-based screening were independently performed using descriptive statistics. General analysis of variance was performed for the main effect of genotype. Similarly, the data for the 7-month field screening was analysed to determine descriptive statistics, including mean ($\bar{\text{x}}$) and range, followed by a general analysis of variance of genotype and block main effect and genotype-by-block interactive effects. Multivariate analysis of trait space was carried out on the 7-month field screening data employing principal components analysis (PCA) to identify major traits accounting for most of the variation among the studied field-grown cassava genotypes. The PCA was based on the correlation matrix and the number of significant principal components was determined based on the eigenvalue-one criterion, retaining any component with an eigenvalue greater than one [19, 25, 26]. Following the PCA, the squared cosine (cos^2^) was computed which gave the quality of representation of the variables on the factor map and the total contribution of individual traits (contrib). Analysis of variance was also conducted on yield and yield-related data measured on the mature (12 months old) field-grown plants for both 2017 and 2018. Pearson’s correlation coefficients for pairs of the shoot and/or root traits were calculated for plants sampled on pot-grown 30 and 45 DAP juvenile plants, as well as on 7- and 12-month field-grown plants at a level of 5%. Subsequently, simple linear regression models were fitted on significantly correlated traits and where multiple traits at the juvenile stage were significantly correlated with a trait at maturity, Generalized Linear Model (GLM) was applied for multiple regression. For the mature plants’ data, broad-sense heritability (*H*^*2*^) across years was estimated as the quotient of the estimated variance associated with the genotypic effect and the total phenotypic variance for a given trait (σg2 /σp2) [23, 27]. The phenotypic variance was calculated using Eq (1) as applied by [23]:

$$\sigma_{p}^{2}=\sigma_{g}^{2}+\frac{\sigma_{g}^{2}\times y}{n}+\frac{\sigma_{\varepsilon }^{2}}{rn}$$ (1)

where: r is the number of replicates, n is the number of years and σg^2^ × y is the genotype x year variance.

All ANOVA were performed using GenStat (GenStat Release 12.1, VSN International, Oxford, UK). The FactoMineR and the ‘corrplot packages in the R software, the Language and Environment for Statistical Computing [28, 29, 30] were used for PCA and correlation graphics. Regression models were fitted in Microsoft excel and subsequently, multiple regression was conducted in GenStat (GenStat Release 12.1, VSN International, Oxford, UK). The package Factoextra was used for the visualization of cos^2^ and the contrib results [28].

## 3.0 Results

### 3.1 Genotypic variation in traits of juvenile plants

**Fig 2:**
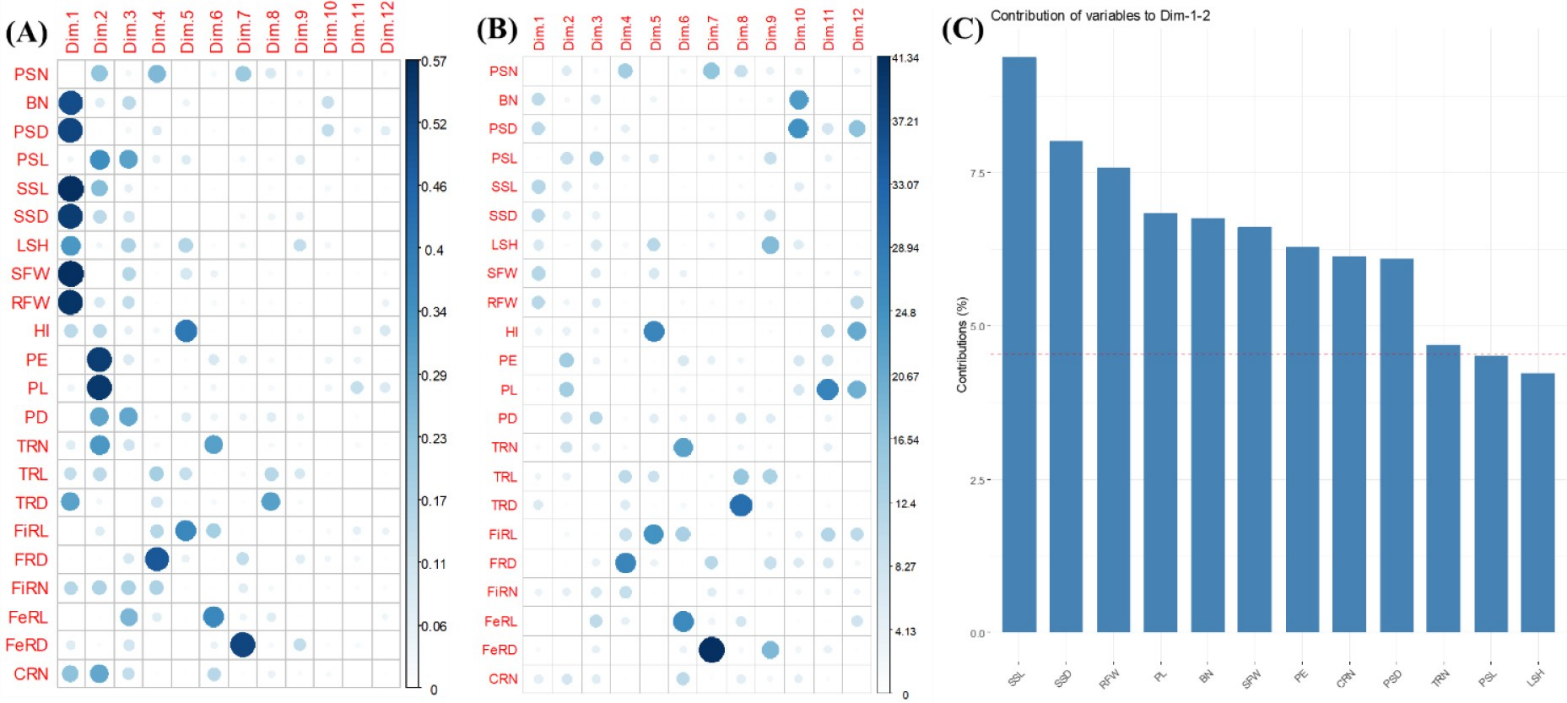
**A:** Plot of quality of representation of the variables (cos^2^ of variables) on the factor map for all dimensions, including the first seven dimensions considered significant following the PCA. Components with eigenvalues greater than one were considered significant in PCA. **B:** Plot showing the total contribution of variables in accounting for the variability in the principal components. **C**: Plot showing the total contribution of variables in accounting for the variability in PC1 and PC2. The red dashed line on the graph indicates the expected average contribution and variables with a contribution greater than this expected average could be considered as important.

Significant differences between genotypes were found for most of the traits examined (14 of the 18 traits examined at both 30 and 45 DAP; *p*<0.01; S1 Table). For some traits, the ranking of genotypes was however not consistent for the two sampling dates (S1A-S1H Fig). The BdBR varied from 1.13±0.125 (F6) to 1.61±0.111 (H8) and from 0.69± 0.23 (B2) to 1.50±0.10 (H8) for 30 and 45 DAP, respectively (S1A Fig). The BdUNR varied from 1.087± 0.0642 (C3) to 1.733± 0.071 (H8) and from 0.478± 0.19 (C3) to 1.367± 0.08(H8) for 30 and 45 DAP, respectively (S1B Fig). There was no genotypic variation in specific root length (SRL) but SRL recorded on 30 DAP were significantly lower (*p*<0.001) than that recorded on 45 DAP (Fig 2C). There was a significant difference in diameter of basal roots at both 30 DAP (*p* <0.05; S1E Fig) and 45 DAP (*p* <0.001; S1E Fig) but the diameter of lower nodal roots only differed significantly between the eight genotypes at 30 DAP (*p* = 0.003; S1F Fig). Genotype H8 was superior in majority of traits, recording the highest BdBR (S1A Fig), BdUNR (Fig 2B), TRL (S1D Fig) and TNR at 30 DAP (S1G Fig). At 30 DAP, SFW ranged from 8.3±0.81 (B2) to 31.5±3.2 (G7), while at 45 DAP, the same trait varied from 22.8±5.2 (C3) to 42.3±4.6 (A1) (S1H Fig). Fresh root biomass also varied from 9.0±2.15 (E5) to 20.83±3.8 (A1) at 30 DAP but ranged between 10.08±1.58 (C3) to 25.83±3.6 (A1) at 45 DAP (S1H Fig). As expected, there were significant differences (*p*<0.05) between measurements on 30 and 45 DAP, except for upper nodal roots, specific root length, and basal root length (S1 Fig and S1 Table).

### 3.2 Field screening at 7 and 12 months after planting

#### 3.2.1 Variation in 7 months’ field-grown genotypes

Significant phenotypic variation (*p*<0.05 or *p*<0.001) was observed for 18 of the 22 shoot or root traits examined in plants sampled at 7 MAP (S2 Table). Largely, variation between blocks was significant for a few traits and block-by-genotype interaction was also rare (S2 Table). The minimum value for some traits was zero and for such traits, there was a greater range of variation. These traits included branch level number (BN), commercial roots number (CRN), feeder roots-, fibrous roots-, and peduncle-, as well as secondary stem-related traits (S2 Table). Among the traits which recorded non-zero minimum values, traits with a 10-fold or greater range of variation included root fresh weight and shoot fresh weight, for which there were approximately 13 and 30-fold range of variation, respectively (S2 Table). One of the more broadly variable root architectural traits was tuberous roots number, for which the maximum value was 7.5 times greater than the minimum value. Phenotypic variation was also observed for primary stem length, tuberous roots diameter, and tuberous roots length, as well as leafless stem height and harvest index (HI) with approximately 8, 2, 3, 4 and 1-fold range of variation, respectively (S2 Table).

Shoot fresh weight varied from 0.95± 0.14g (7G) to 3.47± 0.41g (1A), while RFW varied from 1.65±0.36 (7G) to 4.12±0.4 (1A) (S2A Fig). The study indicated a tendency of root partitioning efficiency in genotype 5E as it had one of the highest RFW and the highest HI of 0.697±0.04 (S2A Fig) and yet recorded the least primary stem length (50.8±13.76 cm; S2C Fig). Again, while genotype 5E may be partitioning root biomass into larger root mass, genotype 8H produced many roots of various categories (Fig 2B). Secondary stem length varied from 10.7±6.77cm (7G) to 92.5±0.92cm (3C), while the length of tuberous roots ranged from 25.4±3.0cm (7G) to 38.2±3.8cm (6F) (S2C Fig). Tuberous root diameter varied between 43.5±2.0 and 69.4±5.4, with TRD ranking in the order: 6F< 5E< 7G< 3C< 1A< 2B< 8H< 4D (S2D Fig). The diameters of peduncle and primary stem varied from 0 (3C) for genotypes without peduncles to 11.05±3.0 (1A), and from 12.48±2.9 (7G) to 22.19±0.2 (3C), respectively (S2D Fig).

#### 3.2.2 Multivariate analysis of traits of 7-month field-grown cassava plants

Fig 2A shows the quality of representation of the variables (cos^2^ or squared coordinates) on the factor map for dimensions, including those considered significant following the PCA. Traits including BN, PSD, SSL, SSD, fSFW and fRFW were well represented on PC1 with cos^2^ between 0.46–0.57 (Fig 2A). Peduncle extent and length were well represented on PC2 with cos^2^ of 0.46–0.57, whilst FiRD and FeRD were respectively well represented on PC6 and PC7 with cos^2^ of approximately 0.55 (Fig 2A). Plot highlighting the most contributing variables for each dimension considered significant after PCA is presented in Fig 2B. The first seven principal components (PCs) with an eigenvalue greater than one explained 78.1% of the total variation for the 22 shoot and root system traits examined for the 7-month field-grown cassava plants (S3 Table and Fig 2B). The first dimension loadings, which accounted for 22% of the variation, largely separated stem- and biomass-related traits (e.g.: primary stem diameter, secondary stem length and diameter, leafless stem height, fresh shoot and root biomass) from other traits (S3 Table and Fig 2B). The relative magnitude of eigenvectors for PC2 was 17.4%, explained mostly by the peduncle- and tuberous root number-related traits (S3 Table and Fig 2B). One (feeder roots number), three (primary stem number, tuberous roots length and fibrous roots diameter), two (fibrous roots length and harvest index), one (feeder roots length) and one (feeder roots diameter) traits were significantly correlated to the third to seventh dimensions, with contributions of 11.5, 8.1, 6.9, 6.5 and 5.8%, respectively (S3 Table and Fig 2B). Ten traits, including SSL, SSD, fRFW, PL, BN, fSFW, CRN and TRN contributed to the variability in the first two dimensions (Fig 2C).

#### 3.2.3 Variation, variance components and broad-sense heritability estimates in 12 months’ field-grown cassava genotypes

Combining the data for the 2017 and 2018 screening, significant differences (*p* = 0.001) was realized in SFW between the eight cassava genotypes (S3A Fig) but no variation was observed in SFW between years in the field-grown mature plants. The fresh shoot biomass of the eight genotypes showed about 1.2-fold difference, ranking in the order: 4D< 7G< 8H< 1A< 6F< 2B< 5E< 3C (S3A Fig). Significant differences (*p* = 0.037) was realized in RFW between eight genotypes. While there was no year effect (*p* = 0.552), significant interaction (*p* = 0.007) between genotype and year was observed on RFW (S3B Fig). There was genotype (S3C Fig) and genotype-by-year effect on HI (*p*<0.001) but there was no significant variation (*p* = 0.536) in HI between the two years (S3D Fig). The HI at maturity ranged from 0.37±0.03 (3C) to 0.60± 0.02(8H) (S3C Fig). The number of commercial or marketable roots significantly varied (*p* <0.001) between the genotypes with over 100% increase between the minimum mean CRN (≈3 roots for 7G) and the maximum mean CRN (≈6 roots for 8H) (S3E Fig). The difference in the number of commercial roots recorded for the two years screening was insignificant (*p* = 0.643), although there was significant (*p* <0.001) genotype-by-year interaction (S3F Fig). A similar observation was made in the analysis of the total number of roots, which comprised of the sum of commercial, non-commercial and feeder roots (Fig 6G and 6H). The effects of genotype and the interaction between genotype x year of trial x block accounted for most of the experimental variation (Table 2). The effect of genotype alone ranged from 3.2% for root fresh weight to 43.6% for shoot fresh weight (Table 2). Broad-sense heritability (*H*^*2*^) estimates were generally intermediate to high. The *H*^*2*^ was highest (0.87) for the number of commercial roots, followed by (0.78) for shoot fresh weight. Root fresh weight had the least H^2^ (0.45; Table 2).

**Table 2:** Estimates of variance components and broad-sense heritability (*H*^*2*^) for yield and yield-related traits measured in 12-months’ field-grown, mature cassava plants in 2017 and 2018.

Trait (Unit)	Variance components (%)								
	Genotype	Block	Year	Gen. x Blk.	Gen. x Year	Blk. x Year	Gen. x Blk. x Year	Residual	Broad sense *(H*^*2*^*)*
Fresh weight of roots (kg)	3.2	78.5	0.0	0.0	9.6*	0.0	0.0	8.7	0.45
Fresh weight of shoot (kg)	43.6*	0.0	0.0	0.0	20.8	2.2	0.0	33.4	0.78
Harvest index at maturity	2.5**	1.5	65.0	0.0	7.3**	0.0	1.4	22.3	0.58
Number of commercial roots	23.4**	3.5	0.0	0.0	4.3**	0.0	0.3	68.5	0.87
Total number of roots	14.1**	1.2	0.0	0.0	27.8*	0.0	0.0	56.8	0.60

### 3.3 Comparison of juvenile and mature cassava traits

**Fig 3:**
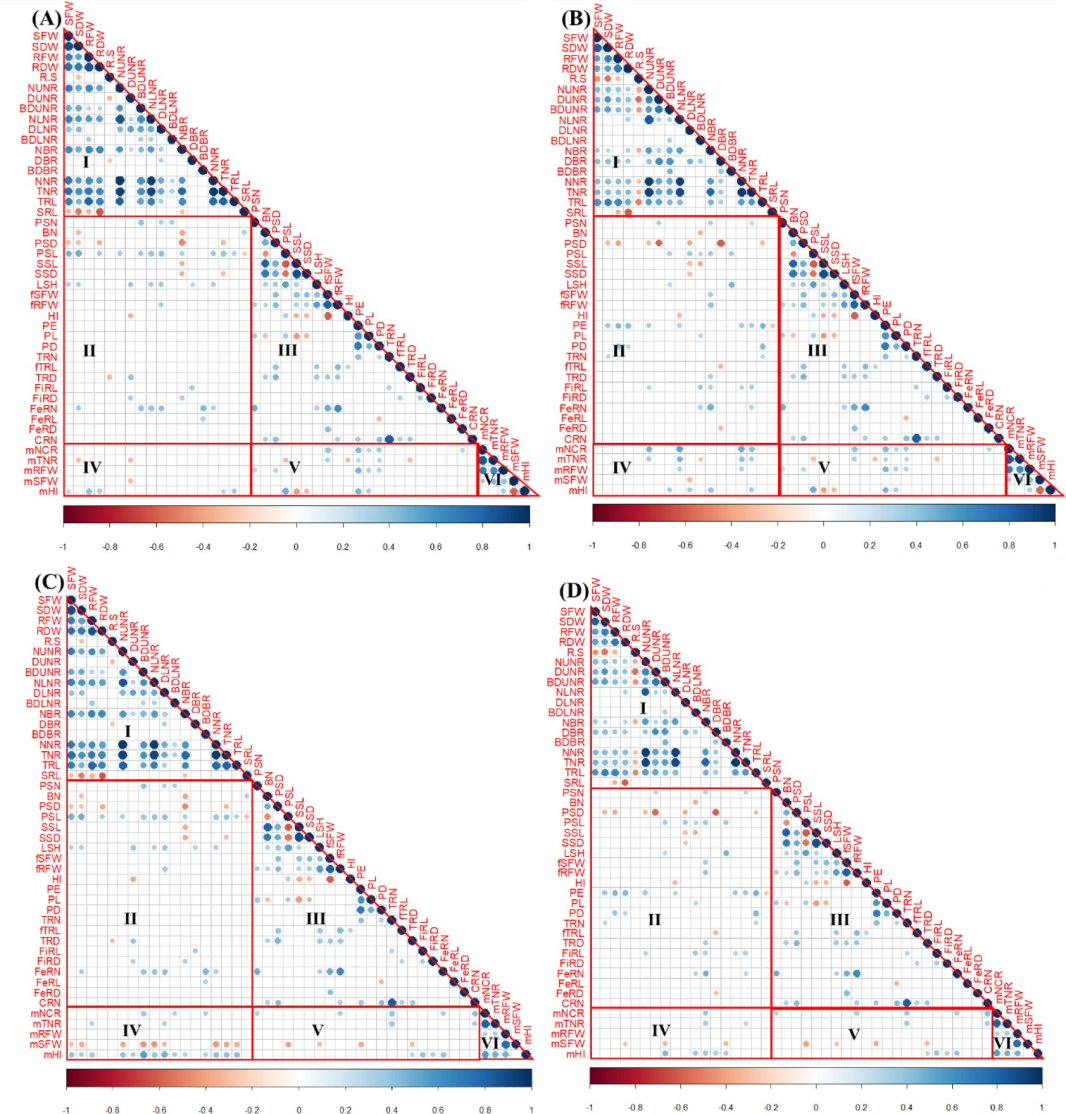
Phenotypic correlations between traits observed in cassava plants grown in soil-filled pots for 30 or 45 days after planting (DAP) and in the field for 7 or 12 months. **A**: correlations between traits measured in 30-DAP-pot grown, 7-months plants and 12-months-field grown plants in 2017; **B**: correlations between traits measured in 45-DAP-pot grown, 7-months plants and 12-months-field grown plants in 2017; **C**: correlations between traits measured in 30-DAP-pot grown plants, 7-months plants and 12-months-field grown plants in 2018; and **D**: correlations between traits measured in 45-DAP-pot grown plants, 7-months plants and 12-months-field grown plants in 2018. In each panel, **I, II, III, IV, V** and **VI** are correlations between traits measured in 30/45-DAP plants; 30/45-DAP and 7-months plants; within traits of 7-months plants; 30/45-DAP plants and 12-months plants; 7 and 12-month plants; and within traits of 12-months plants, respectively. Full names of traits shown in the matrix are can be found in Tables 1–4. Size and colour of circles within the matrix indicate the magnitude of correlation. The scale is indicated in the bar below the matrix. Blank boxes indicate non-significant relationships (*p*< 0.05).

Fig 3 shows the correlation for all traits measured at in juvenile plants at the two sampling stages and the field-grown plants both 2017 and 2018 (including sampling at 7 MAP in 2018). Approximately 53% (81 out of possible 153) and 58% (89 of 153) of all potential correlations were statistically significant (*p* < 0.05), with varying strengths, between traits measured in 30 DAP (Fig 3A-3I and 3C-3I) and 45 DAP (Fig 3B-3I and 3D-3I), respectively. Similarly, approximately 28% (64 of 231), 80% (8 out of possible 10) and 70% (7 of 10) of all potential correlations were statistically significant (*p* < 0.05), with varying strengths, between traits measured in plants harvested at 7 MAP (Fig 3-III), 12 MAP in 2017 (Fig 3A and 3B-VI), and 12 MAP in 2018 (Fig 3C and 3D-IV), respectively.

There were also significant correlations between traits measured in juvenile and matured plants but these correlated traits were generally fewer than the number of correlated traits at a given growth stage. For example, the number of significant correlations between juvenile cassava traits and traits at 7 MAP (Figs 3A-II, 3B-II, 3C-II and 3D-II; 4 and 5) was less than the number of correlated traits at 30 or 45 DAP or 7 MAP. A similar observation applies to the number of correlations between juvenile cassava traits and mature plants (Figs 3A-IV, 3B-IV, 3C-IV and 3D-IV and 6 and 7) and correlations between traits measured in 7 and 12 MAP (Fig 3A-V, 3B-V, 3C-V and 3D-V). Approximately between 12 and 13% of all potential correlations were statistically significant (*p* < 0.05) between traits measured in juvenile plants and those at 7 MAP (Fig 3-II). Similarly, approximately between 9 and 31% of all potential correlations were statistically significant (*p* < 0.05) between traits measured in juvenile and 12-month old-field-grown plants (Fig 3-IV). Traits measured in plants at 7 and 12 MAP also showed significant correlations for approximately between 14 and 19% of all potential correlations (*p* < 0.05; Fig 3-V).

**Fig 4:**
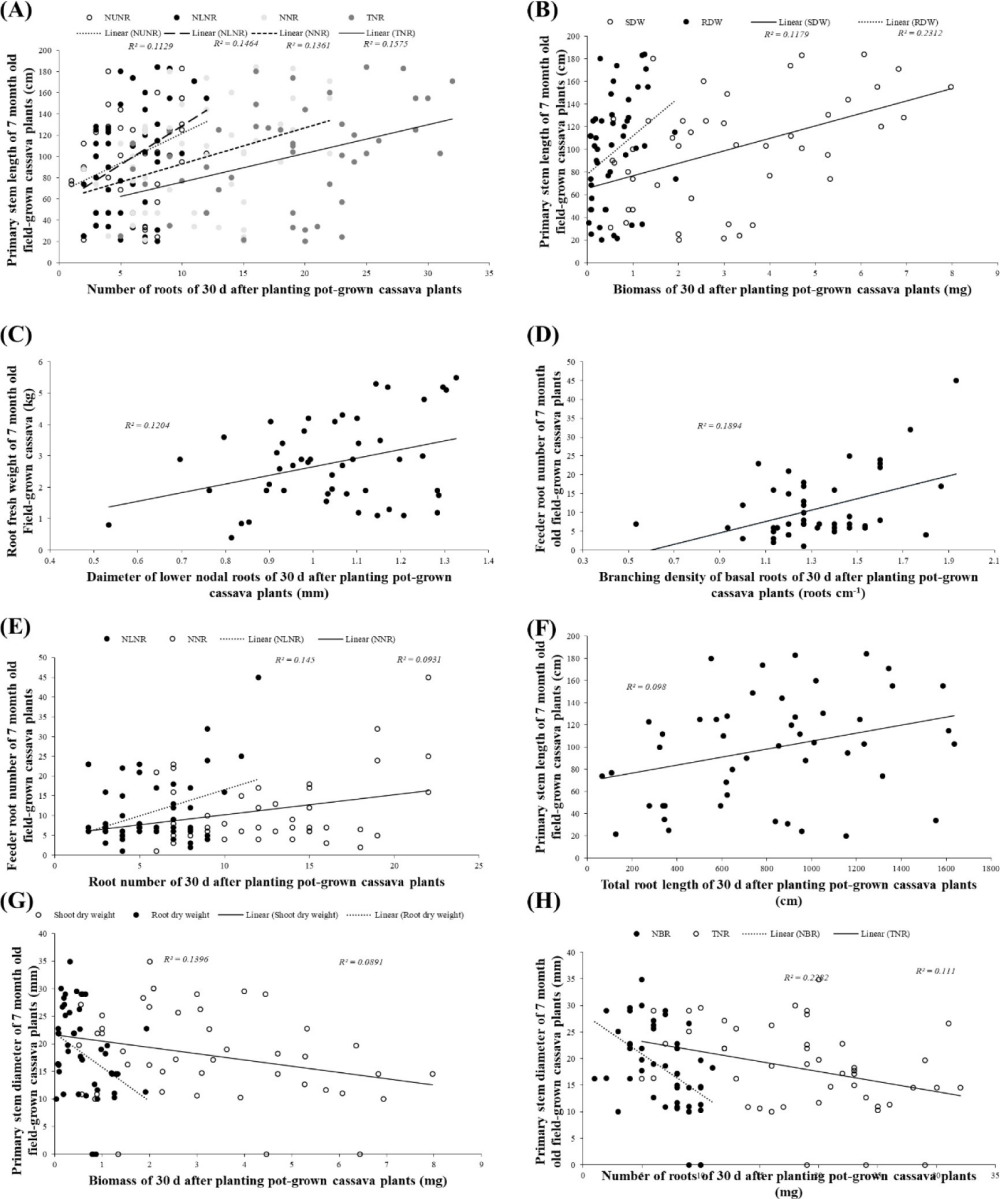
Examples of significant (*p*<0.05) correlations between shoot or root systems traits measured in cassava plants grown in the field for 7 months and traits measured from the same genotypes of cassava grown in soil-filled pots for 30 days. **A**: primary stem length with the number of upper nodal roots (NUNR, *r* = 0.34), number of lower nodal roots (NLNR, *r* = 0.38), number of nodal roots (NNR, *r* = 0.37), and total number of roots (TNR, *r* = 0.40); **B**: primary stem length with shoot dry weight (SDW, *r* = 0.48) and root dry weight (RDW, *r* = 0.34); **C**: root fresh weight with the diameter of lower nodal roots (DLNR, *r* = 0.35); **D**: number of feeder roots with branching density of basal roots (BDBR, *r* = 0.43) **E**: number of feeder roots with the number of lower nodal roots (NLNR, *r* = 0.38) and number of nodal roots (NNR, *r* = 0.43); **F**: primary stem length with total root length (TRL, *r* = 0.31); **G**: primary stem diameter with shoot dry weight (SDW, *r* = -0.30) and root dry weight (RDW, *r* = -0.32) and **H**: primary stem diameter with number of basal roots (NBR, *r* = -0.48) and the total number of roots (TNR, *r* = -0.33).

**Fig 5:**
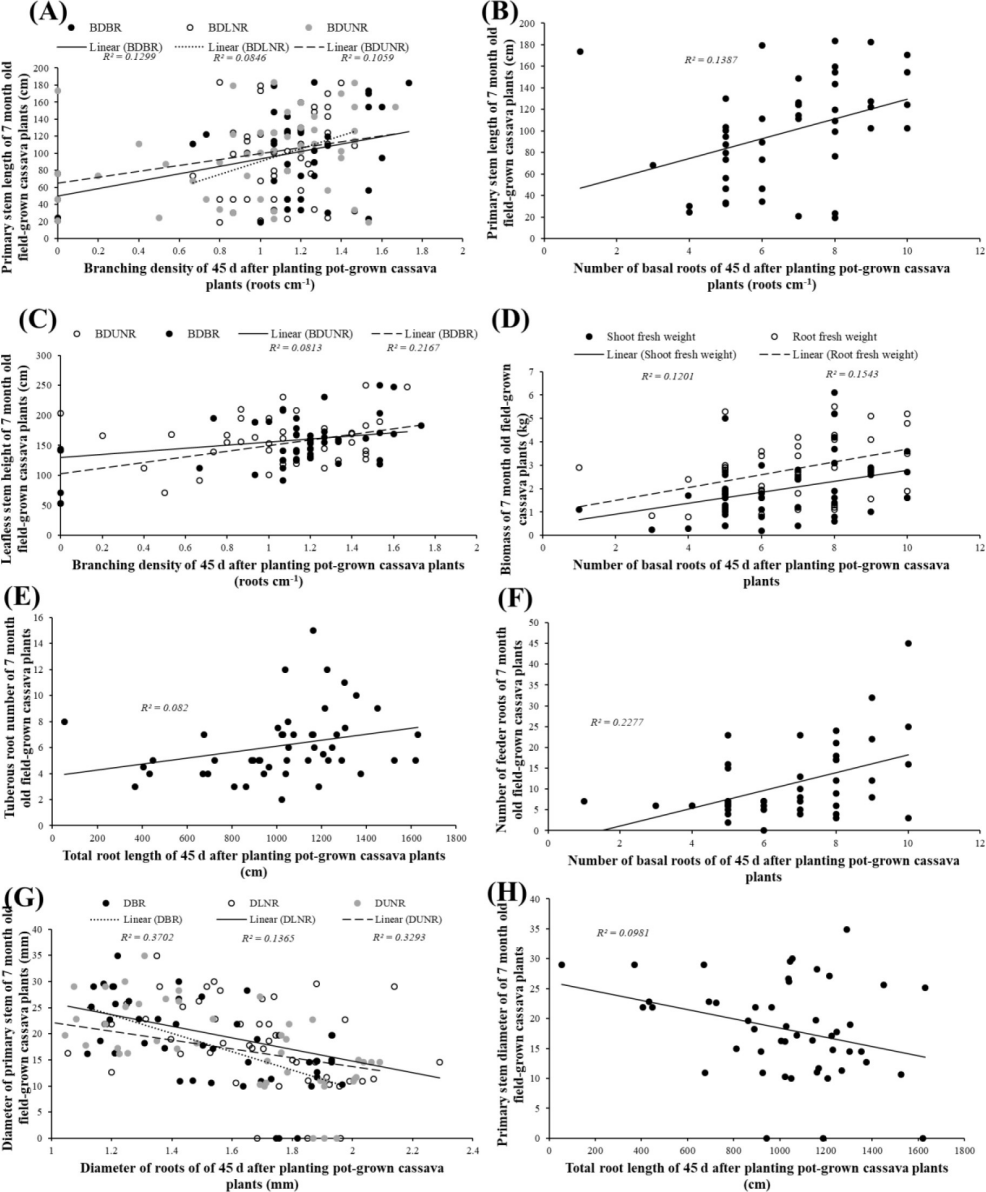
Examples of significant (*p*<0.05) correlations between shoot or root systems traits measured in cassava plants grown in the field for 7 months and traits measured from the same genotypes of cassava grown in soil-filled pots for 45 days. **A**: primary stem length with branching density of basal roots (BDBR, *r* = 0.36), branching density of lower nodal roots (BDLNR, *r* = 0.29) and branching density of upper nodal roots (BDUNR, *r* = 0.33); **B**: primary stem length with the number of basal roots (NBR, *r* = 0.37); **C**: leafless stem height with the branching density of upper nodal roots (BDUNR, *r* = 0.29) and branching density of basal roots (BDBR, *r* = 0.47); **D**: shoot fresh weight with the number of basal roots (NBR, *r* = 0.35), and root fresh weight with the number of basal roots (NBR, *r* = 0.39); **E**: number of tuberous roots with total roots length (TRL, *r* = 0.29); **F**: number of feeder roots with the number of basal roots (NBR, *r* = 0.48); **G**: diameter of the primary stem with the diameter of basal roots (DBR, *r* = -0.61), the diameter of lower nodal roots (DLNR, *r* = -0.37) and diameter of upper nodal roots (DUNR, *r* = -0.57); and **H**: primary stem diameter with total root length (TRL, *r* = -0.31).

**Fig 6:**
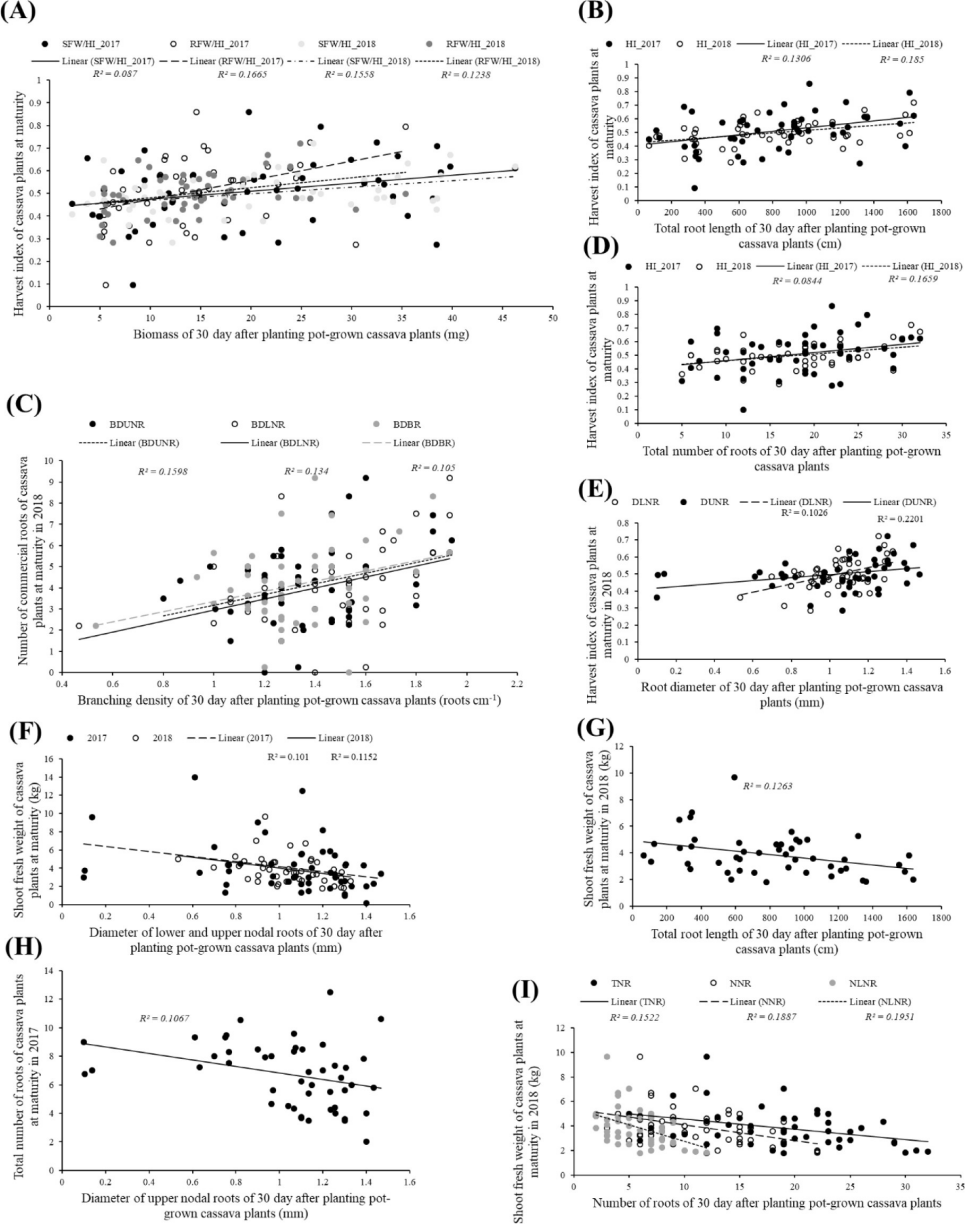
Examples of significant (*p*<0.05) correlations between shoot or root systems traits measured in cassava plants grown in the field for 12 months and traits measured from the same genotypes of cassava grown in soil-filled pots for 30 days. **A**: harvest index in 2017 with shoot fresh weight (*r* = 0.29) and root fresh weight (*r* = 0.41); harvest index in 2018 with shoot fresh weight (*r* = 0.39) and root fresh weight (*r* = 0.35); **B**: harvest index in 2017 with total root length (*r* = 0.36) and harvest index in 2018 with total root length (*r* = 0.41); **C**: number of commercial roots in 2018 with branching density of upper nodal roots (*r* = 0.37), branching density of lower nodal roots (*r* = 0.30) and branching density of basal roots (*r* = 0.32); **D**: harvest index in 2017 with total number of roots (*r* = 0.29) and harvest index in 2018 with total number of roots (*r* = 0.41); **E**: harvest index in 2018 with diameter of lower nodal roots (*r* = 0.47) and diameter of upper nodal roots (*r* = 0.32); **F**: shoot fresh weight in 2017 with diameter of upper nodal roots (*r* = -0.32) and shoot fresh weight in 2018 with diameter of upper nodal roots (*r* = -0.39); **G**: shoot fresh weight in 2018 with total root length (*r* = -0.36); **H**: total number of roots in 2017 with diameter of upper nodal roots (*r* = -0.33); shoot fresh weight in 2018 with total number of roots (*r* = -0.39), number of nodal roots (*r* = -0.43) and number of lower nodal roots (*r* = -0.44).

**Fig 7:**
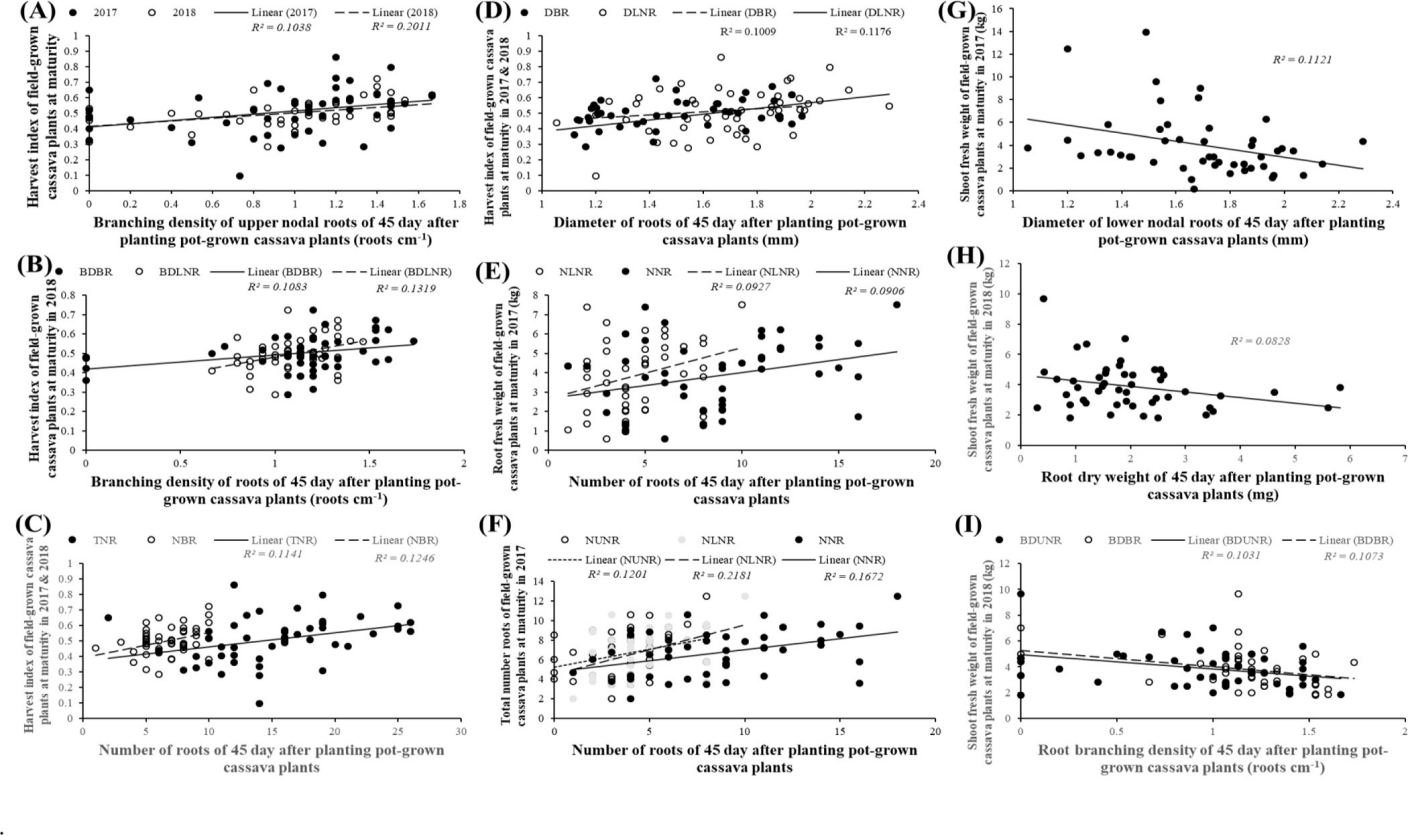
Examples of significant (*p*<0.05) correlations between shoot or root systems traits measured in cassava plants grown in the field for 12 months and traits measured from the same genotypes of cassava grown in soil-filled pots for 45 days. **A**: harvest index in 2017 with branching density of upper nodal roots (*r* = 0.32) and harvest index in 2018 with branching density of upper nodal roots (*r* = 0.45); **B**: harvest index in 2018 with branching density of lower nodal roots (*r* = 0.36) and with branching density of basal roots (*r* = 0.33); **C**: harvest index in 2017 with the total number of nodal roots (*r* = 0.34) and harvest index in 2018 with number of basal roots (*r* = 0.35); **D**: harvest index in 2017 with the diameter of lower nodal roots (*r* = 0.34) and harvest index in 2018 with the diameter of basal roots (*r* = 0.32); **E**: root fresh weight in 2017 with number of lower nodal roots (*r* = 0.30) and with number of nodal roots (*r* = 9.30); **F**: total number of roots in 2017 with number of upper nodal roots (*r* = 0.35), with number of lower nodal roots (*r* = 0.47), and with number of all nodal roots (*r* = 0.41); **G**: shoot fresh weight in 2017 with the diameter of lower nodal roots (*r* = -0.33); **H**: shoot fresh weight in 2018 with root dry weigh (*r* = -0.29), and **I:** shoot fresh weight in 2018 with branching density of upper nodal roots (*r* = -0.32), and with branching density of basal roots (*r* = -0.33).

Simple linear regression models of selected significant correlations between juvenile and 7 MAP, and between juvenile and 12 MAP are presented in Figs 4–7. A simple linear model of the number of roots (Fig 4A), plant biomass (Fig 4B) and TRL (Fig 4F) measured in 30-day old juvenile plants, with primary stem length from the 7 MAP as the dependent variable, produced a significant positive relationship. Other positive relationships between 30 DAP and 7 MAP traits included DLNR and RFW (Fig 4C), branching density of basal roots and number of feeder roots (Fig 4D and 4E). Biomass (Fig 4G) and root number (Fig 4F) of 30 DAP plants produced a significant negative relationship with the primary stem length of 7 MAP.

A linear model of branching density of different root categories in 45 DAP showed a significant positive relationship with primary stem length (Fig 5A) and leafless stem length (Fig 5C) of plants at 7 MAP. Other positive relationships between 45 DAP and 7 MAP traits included the number of roots and primary stem length (Fig 5B), number of basal roots and biomass (Fig 5D) and number of feeder roots (Fig 5F), as well as TRL and tuberous root number (Fig 6E). The diameter of roots of plants at 45 DAP produced a significant negative relationship with the primary stem length of plants at 7 MAP (Fig 5G). Total root length at 45 DAP also produced a significant negative relationship with the primary stem length at 7 MAP (Fig 5H).

Pearson’s correlation coefficient analysis of juvenile and mature plant traits revealed intermediate positive and negative significant correlations between some traits measured in 30 DAP and yield or yield-related traits measured in the plants at 12 MAP. Significant (*p*<0.05) correlation coefficients (*r*) for 30 DAP and mature plant traits in 2017 ranged between -0.33 to -0.30 and 0.29 to 0.41. Significant (*p*<0.05) *r* values for 30 DAP and mature plant traits in 2018 were between -0.44 to -0.30 and 0.29 to 0.55 (Fig 3A and 3B). Most 30 DAP traits which correlated with mature-plant traits measured in 2017, also correlated with same mature-plant traits measured in 2018 (Figs 3A, 3B and 6). For example, biomass (Fig 6A), total root length (Fig 6B) and the total number of roots (Fig 6D) positively correlated with both 2017 and 2018 harvest index at maturity. Similarly, the diameter of nodal roots consistently had a negative significant correlation with both 2017 and 2018 fresh shoot biomass (Fig 6F). There were some traits measured in 30 DAP plants that correlated with mature plants traits only in one of the trial years. These included branching density at 30 DAP and number of commercial roots at maturity in 2018 (Fig 6C), root diameter at 30 DAP and HI at maturity in 2018 (Fig 6E), total root length at 30 DAP and shoot fresh biomass at maturity in 2018 (Fig 6G) and diameter of upper nodal roots at 30 DAP and the total number of roots at maturity in 2017 (Fig 6H). Although many traits measured from 30 DAP juvenile plants significantly correlated with traits of mature field-grown cassava plants, the percentage of variation in the response variable explained by the linear regression model for the 30 DAP versus mature plant traits was between 8.4 and 22.0% (Fig 3A–3I).

Multiple regression improved, albeit slightly, the percentage of variation in the response variable explained by the models. From a Generalized Linear Model (GLM), traits measured in plants at 30 DAP explained 11.4% (*p = 0.011*) of the variation in HI mature plants at 12 MAP in the field in 2017. Similarly, for 2018, approximately 36% (*p* = 0.002) of the variation in HI of mature plants was explained by the traits measured in juvenile plants. Also, a GLM of BDUNR + BDUNR.BDLNR + BDBR measured in plants at 30 DAP explained 17.4% (*p* = 0.01) of the variation in NCR in plants at 12 MAP in 2018.

Significant (*p*<0.05) correlation coefficients for plants at 45 DAP and mature plant traits in 2017 ranged between -0.33 to -0.29 and 0.30 to 0.52. Significant (*p*<0.05) correlation coefficients for 45 DAP and mature plant traits in 2018 were between -0.33 to -0.29 and 0.29 to 0.45 (Fig 3C and 3D). Most of 45 DAP traits which correlated with mature-plant traits measured in 2017, also correlated with same mature-plant traits measured in 2018 (Figs 3C, 3D and 7). For example, the branching density of upper nodal roots (Fig 7A), number of roots (Fig 7C) and diameter of roots (Fig 7D) positively correlated with both 2017 and 2018 harvest index at maturity. Some traits measured in 45 DAP plants correlated with mature plants traits only in one of the field trials. These included, branching density of basal and lower nodal roots at 45 DAP and HI at maturity in 2018 (Fig 7B); the number of roots at 45 DAP and RFW (Fig 7E) in 2017; and the number of roots at 45 DAP and a total number of roots at maturity in 2017 (Fig 7F). Fig 7G–7I show examples of significant negative correlations between 45 DAP and mature plant traits. Although many traits measured from 45 DAP juvenile plants significantly correlated with traits of mature field-grown cassava plants, the coefficient of determination from the simple linear regression models, which ranged from 0.083 to 0.22 (Fig 7A–7I), were small.

Some traits measured in the 45 DAP juvenile plants explained 20.5% (*p* = 0.011) of the variation in the HI of mature plants at 12 MAP in 2017. Also, a GLM of some traits measured at 45 DAP, with the number of commercial roots (NCR) from mature field-grown plants in 2017, produced a highly significant (*p*<0.001) positive relationship (NCR in field-grown plants = 3.469±0.707 + 0.409±0.233*NUNR (45 DAP) + 0.585±0.216*NLNR (45 DAP) + -0.226±0.113*TNR (45 DAP)), explaining 29.4% of the variation in NCR in the 2017 field screening. Similarly, a GLM of NUNR, NLNR, NNR, TNR measured in the 45 DAP with the total number of roots in mature 12-month field-grown plants in 2017 produced a significant (*p* = 0.003) relationship and explained 24.1% of the variation in TNR in 2017. In the 2018 data, a significant (*p* = 0.003) positive relationship which explained 26.0% of the variation in HI of field-grown plants was observed when the following terms: Constant + BDUNR + BDLNR + BDBR + BDLNR.B DBR + NBR (45 DAP), were fitted against HI as the response variate. Again, GLM of root-number-related traits at 45 DAP (NBR+TNR+NBR.TNR) with the total number of roots of mature plants measured in 2018 was significant (*p* = 0.16) and explained 15.4% of the variation in TNR of field-grown plants.

## 4.0 Discussion

### 4.1 Genetic variation and robustness of phenotyping approach

The identification of superior genotypes based on multiple traits is a key objective of cassava improvement trials. In root crops, the ability to identify these genotypic differences, especially in root system architecture, at the juvenile stage could substantially reduce the cost and time of selection and breeding efforts. This could be aided by a robust, inexpensive and rapid phenotyping approach. In the current study, multiple traits, including those said to be important for resource capture in cassava [6, 16, 22], were measured. At the juvenile stage, we adopted the root categorisation presented in [14] and [16] to divide the cassava roots into three classes, namely, upper nodal, lower nodal and basal roots. Previously, evidence was provided that well-developed branching pattern, including the number and lengths of nodal and basal roots, were associated with resource capture in cassava [16]. The results in the current study showed significant genotypic variations in many of the traits measured from both the juvenile plants (S1 Fig) and plants at 7 months after planting (MAP) (S2 Fig) and 12 MAP (S3 Fig). At maturity, the differences found were consistent across the two years of screening. Evidence has been provided of similar genetic variation for juvenile traits among cassava lines [31]. Indeed, [14] reported of similar root genotypic variation in total root length, root numbers, root diameters and root branching density in the same panel of cassava cultivars used in the present study. This suggests that our simple, inexpensive phenotyping approach to characterize cassava RSA at the juvenile stage is robust and could provide a time-saving and less laborious option for a multi-trait selection in breeding for improved genotypes in cassava. The simple phenotyping approach adopted in this study helps obviate several constraints to root phenotyping in general and cassava in particular.

Genotype H8 is a white-flesh cultivar called ‘Capevars bankye’. This cultivar, which is highly preferred by farmers, has been released and cultivated since 2005. Consistent with the data in [14], it was ranked among the top-performing lines for traits such as branching density and total root length (S1 Fig). The remaining seven genotypes used in the present study were bred for high carotenoid content and resistance to cassava mosaic disease and five genotypes, namely 1A, 2B, 5E, 6F and 7G, have just been recommended for release in Ghana. Generally, these genotypes were ranked above genotypes 3C and 4D for many traits that were measured at 30 and 45 DAP and, therefore, it is interesting that 3C and 4D have not been endorsed for release. Indeed, poor yield potential of 3C and 4D was more evident in the 12 MAP field experiment when they obtained the least harvest index and shoot fresh weight, respectively (S3 Fig). The significant differences found between the genotypes in the juvenile experiment thus emphasise the importance of screening cassava at the juvenile stage for desirable characteristics, which include both farmer-preferred traits such as yield potential and breeder-preferred traits such as soil resource capture and use efficiency. Certain general trends were evident from the RSA measurements at the juvenile stage. There was a significant difference in most traits between the two sampling dates (S1 Table). While branching density, the number of roots and specific root length decreased from 30 to 45 DAP, total root length and root diameters increased between the two sampling time points (S1 Fig). In [16], similar observations were made where the total number of axile roots increased until 60 DAP, thereafter decreased, except that in the present study, decrease in axile started only after 30 DAP. Since root diameters increased from 30 to 45 DAP, we hypothesize that the abscission of axile roots from the cutting leading to a reduction in root number and density might be a cue for the initiation of storage roots thickening [16]. The initiation of storage root formation in cassava could start as early as 45 DAP [32]. Moreover, the soil used in this study was unamended with any additional nutrients and so it is conceivable that after 30 DAP soil nutrients would have decreased. Reduced root number and branching, in tandem with increasing root length, might be adaptive strategies to reduce the metabolic costs of soil exploration and improve the acquisition of limiting soil resources [33, 34].

### 4.2 Trait contribution to variation

The results of the PCA (S3 Table) suggest that in the panel of cassava genotypes used in the present study, shoot and root-related traits are contributing almost equally to the genetic variability of near-maturity field-grown cassava, and therefore both could be exploited in the breeding of genotypes for targeted environmental conditions and higher harvest index. If the first four PCs, which accounted for approximately 60% of the total variation are considered, then primary stem-related traits (diameter, number, length), secondary stem length and diameter, leafless stem height, fresh shoot and root biomass, peduncle-related traits (length, diameter and extent), tuberous roots-related traits (number and length), feeder roots number, and fibrous roots diameter, were responsible for most of the phenotypic variation in the 7-months old field-grown plants. As was observed in the PC loadings on PC1 and PC2, the results of quality representation showed that BN, PSD, SSL, SSD, fSFW and fRFW were well represented on PC1 with cos^2^ between 0.46–0.57. Traits that are correlated with PC1 and PC2 could be the most important in explaining the variability in the dataset and may be considered in efforts to breed for improved genotypes [14, 23]. Here, 10 traits, including SSL, SSD, fRFW, PL, BN, fSFW, CRN and TRN contributed to the variability in the first two dimensions (S3C Fig), suggesting that these traits are sufficient to differentiate cassava genotypes. Screening for these physiological traits at 7 MAP, could be time- and resource-saving, given that main agronomic evaluation of cassava are commonly conducted at the end of the crop cycle, making the selection process protracted and costly [6].

### 4.3 Components of variance and heritability in 12-months field-grown cassava

Conventional physiological traits measured in studies of cassava at 12 MAP include above and below-ground biomass and number of storage roots [6]. Similarly, these traits were measured at 12 MAP and subsequently computed HI for two years of field evaluation. Reliably estimating variance components and heritability are vital requisites for selection gain and improvement in quantitative traits [23, 35]. Over the two years, the effects of genotype, block and year of trial accounted for most of the experimental variation but various interactions contributed relatively little (Table 2). It was evident that some vagaries in experimental conditions between the two trials might be contributing to the variation in some of the traits examined, in which case, the residual proportion ($\sigma_{\varepsilon }^{2}$) was larger than the genotypic ($\sigma_{g}^{2}$), the block ($\sigma_{b}^{2}$) or the trial ($\sigma_{y}^{2}$) effect (S2 Table). It has been suggested that under conditions of high residual variance, the within-genotype variation might be high and/or there may be the need for a more parsimonious model [23]. Broad-sense heritability was highest for the number of commercial roots (0.87) and shoot fresh weight (0.78) and intermediate for the total number of roots (0.60), harvest index (0.58), fresh weight of roots (0.45) (Table 2). The *H*^*2*^ estimates here are comparable to that reported by Oliveira et al., (2015) for the number of roots (0.51±17) and root and shoot biomass (0.80±0.21) of 12 MAP field-grown cassava under irrigated conditions. Shoot-related traits have been reported to have larger *H*^2^ than root-related traits [27, 36, 37], so it is interesting to note that the number of commercial roots obtained the highest *H*^*2*^ in the present study. The relatively higher heritability estimates for root number and fresh weight of shoot might be indicative of fewer genes controlling these parameters and low environmental influence on the expression of these phenotypes [38, 39], and pointing to the possibility of using simple selection methods for the improvement of these traits in response to stress conditions [39, 40]. This hypothesis is also supported by the fact that the heritability estimate for HI was relatively lower, given that HI is a complex trait computed from multiple traits and therefore is expected to be under the control of several genes.

### 4.4 Relationships between juvenile RSA traits and yield components of mature plants

There is a pressing need to use root phenotyping to assess the productivity of crop plants at an early stage [6]. To this end, we explored the relationships between phenotypic traits measured at the juvenile stage (30 and 45 DAP) and root or yield component traits measured in plants at 7 or 12 MAP. Comparing juvenile plant RSA traits with field traits or field agronomical performance has been reported in several studies, for example in alfalfa [41]; bread or durum wheat [42–45]; common bean [46]; maize [47–49]; oilseed rape [50]; potato [11]; red clover [51] and tomato [52]. The phenotyping technique adopted in the current study allows cost-effective and rapid measurement of cassava root traits, but the roots of juvenile plants growing in pots may not develop exactly as in nature [11, 42, 53]. Moreover, there is no evidence for predicting performance in heterogeneous environments based on a single RSA trait [54]. It has also been suggested that plastic responses of growth linked to the different experimental systems (screen-house versus field), might lead to overly large variations to identify trends in a dataset [12]. Notwithstanding, a systematic root phenotyping system was adopted (Fig 1), over two sampling times (30 and 45 DAP), to provide a useful array of root and shoot traits in juvenile cassava that were related to desirable traits in the mature cassava plants.

Significant correlations (*p<0.05*) between the juvenile RSA traits, and between field-measured traits were observed at 7 MAP (Figs 4 and 5) and between field-measured yield and yield-related traits at 12 MAP (*p<0.05*) (Figs 6 and 7), suggesting that the RSA of 30- or 45 day-old young cassava plants could be explored for predicting field performance of agronomic traits. Moreover, there were several significant, positive inter-traits correlations for a given growth stage than between growth stages in both juvenile and mature plants (Fig 3), suggesting that these correlated traits could be improved simultaneously or some, selected for, as proxies for others [23]. For example, number of lower nodal roots highly correlated with number of upper nodal at 30 DAP (*r* = 0.85) and 45 DAP (*r* = 0.84) and shoot biomass correlated with root biomass at 30 DAP (*r* = 0.68) and 45 DAP (*r* = 0.54). Similarly, at 7 MAP, tuberous root diameter correlated with the diameter of upper nodal roots at 30 DAP (*r* = 0.36); primary stem length with shoot fresh weight at 30 DAP (*r* = 0.46); leafless stem height with branching density of basal root at 45 DAP (*r* = 0.47) and the number of feeder roots correlated with the number of basal roots at 45 DAP (*r* = 0.48) (Fig 4). However, there were negative relationships between some traits. For example, the diameter of upper nodal roots consistently had a negative relationship with the root-to-shoot ratio at both 30 DAP (*r* = -0.32) and 45 DAP (*r* = -0.55), pointing to a positive allometric relationship between the diameter of upper nodal roots and root biomass in cassava.

The root system size of juvenile cassava plants, as represented by the number of roots, diameters, fresh biomass of roots, branching density of different root classes measured in both 30 DAP (Fig 4) and 45 DAP (Fig 5) plants, was positively associated with various traits measured in 7 MAP plants, including primary stem length (Figs 4B and 5A), leafless stem length (Fig 5C), number of feeder roots (Fig 4D and 4E), biomass (Fig 5D) and tuberous root number (Fig 5E). A few inconsistencies found in the correlations between traits measured at 30 and 45 DAP with the traits measured at 7 MAP could be due to some form of stress which was possibly initiated in the plants grown up to 45 days as a result of a decline in soil nutrients in the pots. The present results suggest that an increase in shoot and root biomass in 7-month field-grown plants could be linked to the root system size of younger plants. Evidence has been provided that bigger and vigorous root systems of younger plants are beneficial to acquire more soil resources for early plant growth [42, 55, 56, 57]. Some cassava genotypes, especially early maturing cultivars are harvestable at 7 MAP. [6] suggested that evaluating cassava genotypes at 7 MAP can be used to select good varieties of potentially good yield at 12 MAP. The results of the present study suggest that at the juvenile growth stage, it would be possible to identify genotypes with longer primary stems, more tuberous and feeder roots, as well as increased biomass, which is some of the main characteristics associated with an improved final yield of cassava in the field.

The best GLM for HI for measurements taken at 30 DAP included some consistent traits including fresh shoot and root biomass, as well as traits related to root system size such as total root length and the total number of roots, for both the 2017 and 2018 datasets and explained up to 36% of the variation in HI of 12 MAP field-grown plants. The best GLM for HI for measurements taken at 45 DAP included some consistent traits including branching density of upper nodal roots and number of basal roots, for both the 2017 and 2018 datasets and explained up to 26% of the variation in HI of 12 MAP field-grown plants. Similarly, the best GLM for the total number of roots at 12 MAP for both 2017 and 2018 consistently included the total number of roots from measurements taken at 45 DAP, suggesting that many root traits at the early growth stage are associated with the number of tubers and yield at maturity. Overall, GLMs for HI for measurements taken at the juvenile stage of growth included fresh shoot and root biomass, branching densities and numbers of upper, lower and basal roots and diameter of lower nodal roots, suggesting that root production and size at the juvenile stage are important determinants of economic yield at maturity. Basal roots, for example, might be vital for water uptake and anchorage while nodal roots originating from the nodes of the propagated cutting and spreading horizontally might be more important in nutrient acquisition and tuberization [12].

### 4.5 Utility in cassava improvement programmes

There are effectively four main factors of the genetic gain equation (*ΔG*) that influence breeding progress [58]. These include the phenotypic variation in the population (*σ*_*p*_), the heritability (*h*^*2*^), and selection intensity (*i*). By increasing *σ*_*p*_, *h*^*2*^ or *i*, *ΔG* can be improved but this must be concomitant with a decrease in the last factor, *L*, which describes the length of time necessary to complete a cycle of selection [58]. The present study could be instrumental in the efforts to decrease *L* because selection based on juvenile plant traits could determine how quickly generations can be completed and how many generations can be completed per any given time. [59] noted that selection from juvenile traits can be an efficient method for acquiring *ΔG* while minimizing rotation time. The present work could also have utility in indirect selection for storage root yield, where selection is (i) based on the correlation between traits of the juvenile plants and same traits of mature plants or (ii) applied on another trait as that which shall be improved [60]. It is, however, worth noting that certain prerequisites are important to achieve this benefit. Based on the mathematical rule for the standardized selection response, the success of the indirect selection is dependent on the presence of a genetic correlation between two different traits (correlated response) [60]. Indirect selection can only be advantageous if an indirect character has a greater heritability than primary character, and the genetic correlation between primary and indirect characters is high [60]. Also, for indirect selection to be worthwhile in breeding programmes, it should be easier to measure the indirect character more accurately than it is to measure the primary character and the *i* of the indirect character must also be much greater than that of the primary character [58, 60].

The present protocol can be applied to cassava populations to identify markers which segregate with specific rooting characteristics and ultimately reduce the lengthy time involved in breeding for improved genotypes in cassava. Going forward, it would be ideal to use genetic analysis, which shows closely linked genes or pleiotropy, to confirm the phenotypic correlations of fresh biomass, branching density and diameter-related traits, root length and numbers with yield of mature field-grown plants. This could facilitate the identification of traits which are beneficial for improved resource acquisition and use-efficiency under field and stressed conditions and also enhance the potential of introgressing coincident quantitative trait loci (QTLs) to improve RSA, yield and yield-related traits in cassava.

It must, however, be said that inferences from this paper may be somewhat limited because the genotypes used may not cover the complete natural range of variation in cassava and may not provide full insight into the genetic properties of the species. Thus, the genetic component in this study is more fixed, than random. Ideally, attempts to exploit indirect selection in an applied cassava breeding programme should be based on a random set of genotypes similar to the populations of a conventional breeding programme. It would, therefore, be appropriate to validate our results so that our approach can effectively be applied to actual breeding programmes. There is an ongoing project of using nuclear applications, including gamma rays for mutation induction in cassava, at the Nuclear Agricultural Research, Biotechnology and Nuclear Agriculture Research Institute in Ghana. It is possible to validate our approach using inbred populations of various mutant families, derived from irradiated propagation materials.

While it is imperative to validate our results, indirect selection based on the protocol proposed here could have some improvements over conventional breeding. Conventional cassava breeding is protracted and complex because it involves several stages and many of the traits under selection have a quantitative inheritance with many active genes and strong environmental influence. The accuracy of selection depends on the experimental differences in all of the stages of the breeding programme [61]. We hypothesise that the protocol proposed could be exploited to reduce the duration required in clonal evaluation trials (CET) and even preliminary yield trials (PYT), provided high heritability and genetically correlated traits could be identified in the early growth stage. As part of the ongoing cassava improvement programme, we intend to exploit juvenile traits in CET and/or PYT, alongside the conventional approaches. Hopefully, the genetic value of the clones in the CET phase obtained indirectly through selection from juvenile plants will be a good predictor of the performance of the genotypes in the PYT phase, thereby simplifying the process of variety development.

In a typical breeding programme, thousands of genotypes are involved. The benefit of an indirect selection will be realised if the system is of high throughput, allowing the testing of a much larger number of progenies than otherwise possible so that the selection pressure can be significantly increased. Depending on the experience of the technician, excavation, soaking and cleaning of root systems and measurements of traits required 15 to 18 minutes in the present study. [62] has reported that the soil type in which plants are grown can influence the time needed to uproot and evaluate the roots, suggesting that the time recorded here could be reduced if a more friable soil is used for this work. In the present study, based on the results of the PCA, it may also be feasible to reduce the number of traits to be evaluated to increase the speed of the proposed method. To enable high-throughput phenotyping of the root systems, automated or semi-automated image analysis and low-cost computer vision technologies could be adapted or developed and coupled to the present approach.

## 5.0 Conclusions

There is an urgent need for rapid, inexpensive and robust phenotyping of root system traits of juvenile plants as a basis for identifying genotypic variations and predicting growth and yield performance of mature plants. This can substantially reduce the time, cost and effort for selection and breeding for crop improvement in response to environmental stresses and higher productivity. Despite its global socio-economic importance as a key food security crop, cassava is not easily amenable to such approaches. The results in the current study demonstrate the possibility of applying a simple, rapid, and robust phenotyping approach to identifying important traits in juvenile cassava that can be a basis for predicting field performance of mature plants. Significant genotypic variations were observed in both juvenile and mature plants, and traits with broad-sense heritability were identified. The results provide insights into the dynamics of cassava root and shoot traits, at 30 and 45 DAP and their relationships with traits in 7 and 12 months old (mature) plants. The explanatory power of fresh shoot and root biomass, total root length and the total number of roots measured at 30 DAP as predictors of HI index in mature plants was consistent for both 2017 and 2018. Genotypic differences in some important traits, such as shoot and root fresh weight, in mature plants were consistent for both 2017 and 2018 experiments or screening. The results provide seminal evidence for potentially useful relationships between traits in juvenile and mature cassava plants that can be further explored for predicting harvest index and supporting efforts for improving the crop.

## Supporting information

S1 Fig Genotypic variation in juvenile cassava plants grown in soil-filled pots for 30 and 45 Days After Panting (DAP). **A**: basal roots branching density (30 DAP: *F*_*7,32*_ = *2.50*, *p* = 0.036; 45 DAP: *F*_*7,32*_ = *3.50*, p = 0.007); **B**: upper nodal roots branching density (30 DAP: *F*_*7,32*_ = *7.38*, *p* <0.001; 45 DAP: *F*_*7,32*_ = *5.56*, *p*<0.001); **C**: specific root length (30 DAP: *F*_*7,32*_ = *1.85*, *p* = 0.112; 45 DAP: *F*_*7,32*_ = *0.42*, *p* = 0.881); **D**: total root length (30 DAP: *F*_*7,32*_ = 3.43, *p* = 0.0008; 45 DAP: *F*_*7,32*_ = *3.83*, *p* = 0.004); **E**: basal root diameter (30 DAP: *F*_*7,32*_ = *2.33*, *p* = 0.048; 45 DAP: *F*_*7,32*_ = *6.95*, *p* <0.001); **F:** Lower nodal roots diameter **(**30 DAP:*F*_*7,32*_ = *4.06*, *p* = 0.003; 45 DAP: *F*_*7,32*_ = *2.02*, *p* = 0.084); **G:** total number of roots (30 DAP: *F*_*7,32*_ = *12.74*, *p* <0.001; 45 DAP: *F*_*7,32*_ = *8.51*, *p* <0.001).; total number of roots = number of basal roots + number of lower nodal roots + number of upper nodal roots; **H**: shoot fresh weight (30 DAP: *F*_*7,32*_ = *5.46*, *p*<0.001; 45 DAP: *F*_*7,32*_ = *2.38*, *p* = 0.044); **H**: root fresh weight (30 DAP: *F*_*7,32*_ = *3.15*, *p* = 0.012; 45 DAP: *F*_*7,32*_ = *4.82*, *p* <0.001).
(DOCX)

S2 Fig Genotypic variation in 7-months field-grown cassava plants. **A**: shoot fresh weight (*F*_*7,32*_ = *6.87*, *p* <0.001, root fresh weight (*F*_*7,32*_ = *4.01*, *p* = 0.003), harvest index (*F*_*7,32*_ =, 5.32, *p* <0.001); **B**: number of commercial roots (*F*_*7,32*_ = *6.54*, *p* <0.001), number of tuberous roots (*F*_*7,32*_ = *9.27*, *p*<0.001), number of feeder roots (*F*_*7,32*_ = *12.06*, *p*<0.001); **C**: length of primary stem (*F*_*7,32*_ = *6.92*, *p*<0.001), length of secondary stem (*F*_*7,32*_ = *5.62*, *p*<0.001), length of fibrous roots (*F*_*7,32*_ = *2.60*, *p* = 0.031), length of tuberous roots (*F*_*7,32*_ = *1.96*, *p* = 0.093); and **D**: diameter of peduncle (*F*_*7,32*_ = *5.77*, *p*<0.001), diameter of primary stem (*F*_*7,32*_ = *4.99*, *p*<0.001), dimeter of tuberous roots (*F*_*7,32*_ = *7.09*, *p*<0.001).
(DOCX)

S3 Fig Genotypic variation cassava plants grown in a field for 12-months in 2017 and 2018. **A**: shoot fresh weight (2017 & 2018: *F*_*7,64*_ = *3.33*, *p* = 0.004); **B**: root fresh weight (2017: *F*_*7,32*_ = *2.47*, *p* = 0.038; 2018: *F*_*7,32*_ = *2.51*, *p = 0.035*), **C**: harvest index (2017 & 2018: *F*_*7,64*_ = *10.95*, *p*<0.001); **D**: harvest index (2017: *F*_*7,32*_ = *10.00*, *p*<0.001; 2018: *F*_*7,32*_ = *4.47*, *p* =) 0.001; **E**: number of commercial roots (207 & 2018: *F*_*7,64*_ = *6.33*, *p* <0.001); **F**: number of commercial roots (2017: *F*_*7,32*_ = *5.57*, *p*<0.001; 2018: *F*_*7,32*_ = *5.30*, *p <0.001*); **G**: total number of roots (207 & 2018: *F*_*7,64*_ = *6.10*, *p* <0.001); **H**: total number of roots (2017: *F*_*7,32*_ = *7.28*, *p*<0.001; 2018: *F*_*7,32*_ = *2.52*, *p = 0.035*).
(DOCX)

S1 Table Summary of descriptive statistics and summary of ANOVA results (p-value) of each shoot or root trait by genotype (8 genotypes) from juvenile cassava plants at 30 or 45 days after planting (DAP). The final column shows the ANOVA between traits measured at both 30 and 45 DAP.
(DOCX)

S2 Table Summary of descriptive statistics of the shoot and root traits data and summary of ANOVA (p-value) between field-grown cassava genotypes at 7 months after planting. (DOCX)

S3 Table Results of principal component analysis (PCA) on 22 traits, (8 shoot and 14 root traits) of 8 cassava genotypes grown in the field for 7 months. Loading scores of traits on each component and the proportion of variation explained with the first seven significant components are presented. Components with eigenvalues >1 are considered significant. Bold indicates variable loading scores with the greatest loads on each component. Genotype means data (n = 3) were used for PCA.
(DOCX)
