# Supplementary material for: The search for yield predictors for mature field-grown plants from juvenile pot-grown cassava (Manihot esculenta Crantz)
Source: PLoS One. 2020 May 6;15(5):e0232595. doi: 10.1371/journal.pone.0232595 (PMC7202627; doi:10.1371/journal.pone.0232595)
Supplement: S1 Fig — A: basal roots branching density (30 DAP: F7,32 = 2.50, p = 0.036; 45 DAP: F7,32 = 3.50, p = 0.007); B: upper nodal roots branching density (30 DAP: F7,32 = 7.38, p <0.001; 45 DAP: F7,32 = 5.56, p<0.001); C: specific root length (30 DAP: F7,32 = 1.85, p = 0.112; 45 DAP: F7,32 = 0.42, p = 0.881); D: total root length (30 DAP: F7,32 = 3.43, p = 0.0008; 45 DAP: F7,32 = 3.83, p = 0.004); E: basal root diameter (30 DAP: F7,32 = 2.33, p = 0.048; 45 DAP: F7,32 = 6.95, p <0.001); F: Lower nodal roots diameter (30 DAP:F7,32 = 4.06, p = 0.003; 45 DAP: F7,32 = 2.02, p = 0.084); G: total number of roots (30 DAP: F7,32 = 12.74, p <0.001; 45 DAP: F7,32 = 8.51, p <0.001).; total number of roots = number of basal roots + number of lower nodal roots + number of upper nodal roots; H: shoot fresh weight (30 DAP: F7,32 = 5.46, p<0.001; 45 DAP: F7,32 = 2.38, p = 0.044); H: root fresh weight (30 DAP: F7,32 = 3.15, p = 0.012; 45 DAP: F7,32 = 4.82, p <0.001). (DOCX) [file pone.0232595.s001.docx]

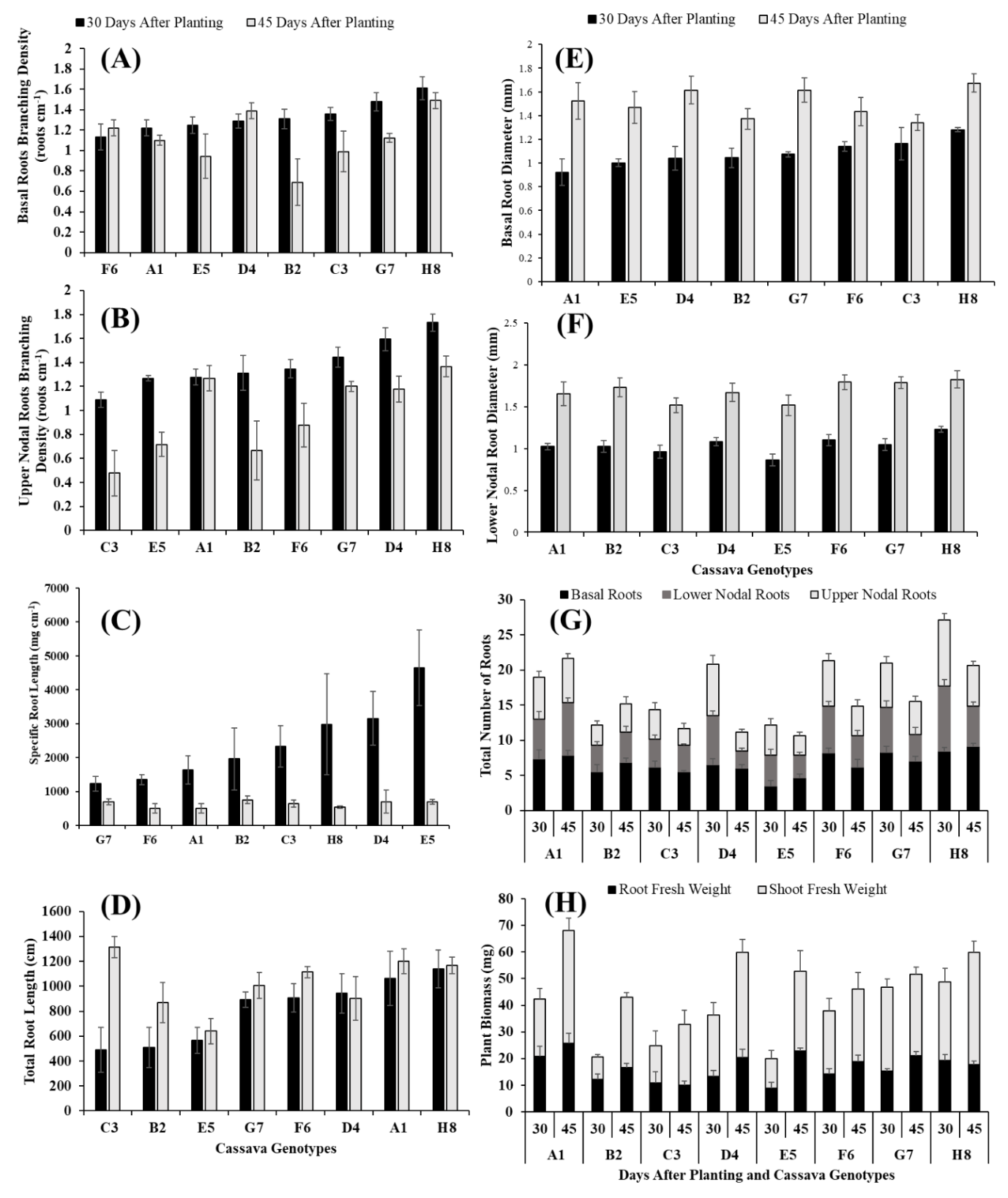


**Supplementary Figure S1**: Genotypic variation in juvenile cassava plants grown in soil-filled pots for 30 and 45 days after panting (DAP). **A**: basal roots branching density (30 DAP: *F_7,32_=2.50*, *p*= 0.036; 45 DAP: *F_7,32_*=*3.50*, p=0.007); **B**: upper nodal roots branching density (30 DAP: *F_7,32_=7.38*, *p* <0.001; 45 DAP: *F_7,32_=5.56* , *p*<0.001); **C**: specific root length (30 DAP: *F_7,32_ =1.85*, *p*= 0.112; 45 DAP: *F_7,32_=0.42*, *p*= 0.881); **D**: total root length (30 DAP: *F_7,32_=*3.43, *p*=0.0008; 45 DAP: *F_7,32_ =3.83*, *p*=0.004); **E**: basal root diameter (30 DAP: *F_7,32_=2.33*, *p*= 0.048; 45 DAP: *F_7,32_ =6.95*, *p* <0.001); **F:** Lower nodal roots diameter **(**30 DAP:*F_7,32_=4.06*, *p*= 0.003; 45 DAP: *F_7,32_*= *2.02*, *p*= 0.084); **G:** total number of roots (30 DAP: *F_7,32_=12.74*, *p* <0.001; 45 DAP: *F_7,32_=8.51*, *p* <0.001).; total number of roots = number of basal roots + number of lower nodal roots + number of upper nodal roots; **H**: shoot fresh weight (30 DAP: *F_7,32_ =5.46*, *p*<0.001; 45 DAP: *F_7,32_=2.38*, *p*= 0.044); **H**: root fresh weight (30 DAP: *F_7,32_=3.15*, *p*= 0.012; 45 DAP: *F_7,32_=4.82*, *p* <0.001).
