# Supplementary material for: The search for yield predictors for mature field-grown plants from juvenile pot-grown cassava (Manihot esculenta Crantz)
Source: PLoS One. 2020 May 6;15(5):e0232595. doi: 10.1371/journal.pone.0232595 (PMC7202627; doi:10.1371/journal.pone.0232595)
Supplement: S2 Fig — A: shoot fresh weight (F7,32 = 6.87, p <0.001, root fresh weight (F7,32 = 4.01, p = 0.003), harvest index (F7,32 =, 5.32, p <0.001); B: number of commercial roots (F7,32 = 6.54, p <0.001), number of tuberous roots (F7,32 = 9.27, p<0.001), number of feeder roots (F7,32 = 12.06, p<0.001); C: length of primary stem (F7,32 = 6.92, p<0.001), length of secondary stem (F7,32 = 5.62, p<0.001), length of fibrous roots (F7,32 = 2.60, p = 0.031), length of tuberous roots (F7,32 = 1.96, p = 0.093); and D: diameter of peduncle (F7,32 = 5.77, p<0.001), diameter of primary stem (F7,32 = 4.99, p<0.001), dimeter of tuberous roots (F7,32 = 7.09, p<0.001). (DOCX) [file pone.0232595.s002.docx]

**
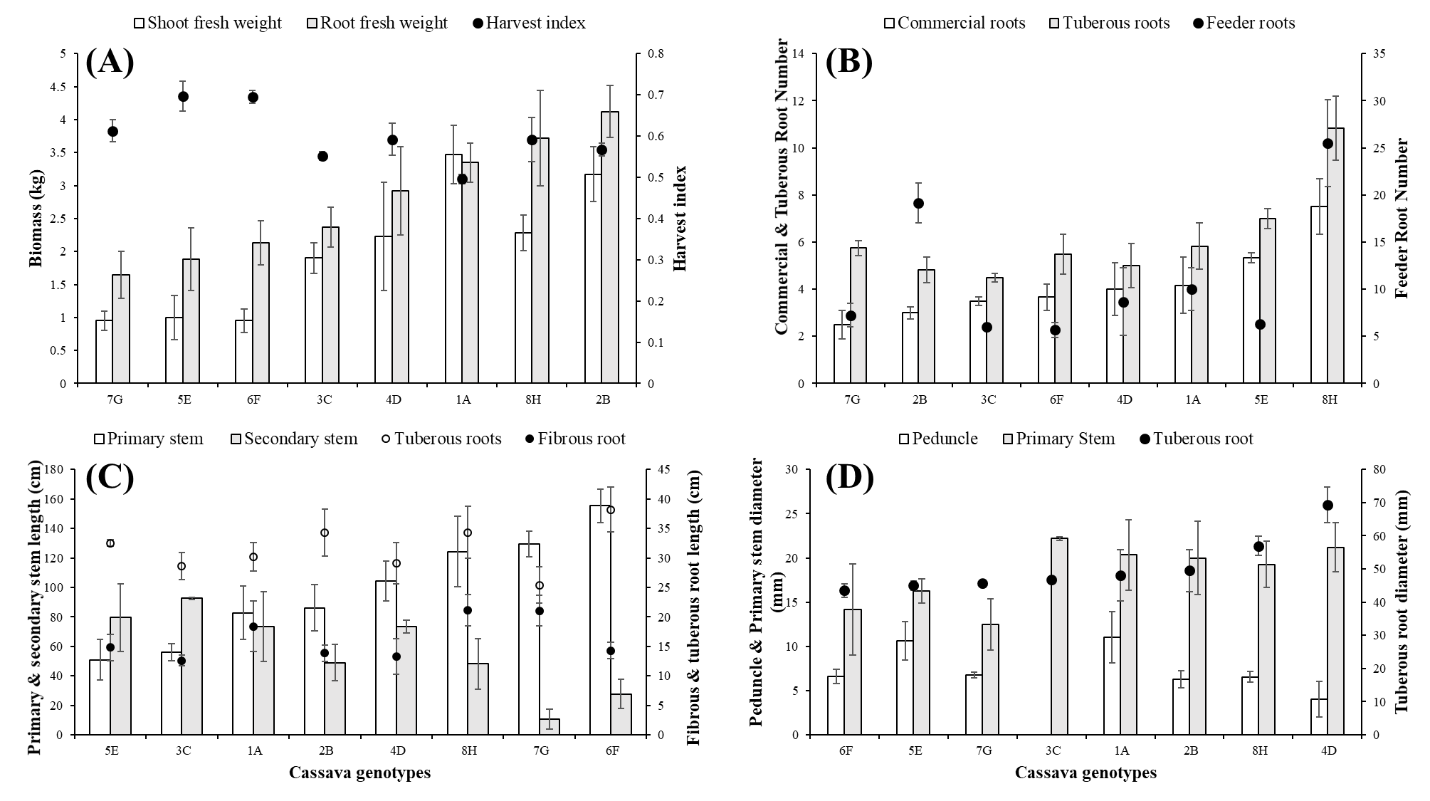
**

**Supplementary Figure S2:** Genotypic variation in 7-months field-grown cassava plants. **A**: shoot fresh weight (*F_7,32_=6.87*, *p* <0.001, root fresh weight (*F_7,32_=4.01*, *p*=0.003), harvest index (*F_7,32_=*,5.32, *p* <0.001); **B**: number of commercial roots (*F_7,32_= 6.54*, *p* <0.001), number of tuberous roots (*F_7,32_=* *9.27*, *p*<0.001), number of feeder roots (*F_7,32_=12.06*, *p*<0.001); **C**: length of primary stem (*F_7,32_=6.92*, *p*<0.001), length of secondary stem (*F_7,32_=5.62*, *p*<0.001), length of fibrous roots (*F_7,32_= 2.60*, *p*= 0.031), length of tuberous roots (*F_7,32_= 1.96*, *p*=0.093); and **D**: diameter of peduncle (*F_7,32_= 5.77*, *p*<0.001), diameter of primary stem (*F_7,32_= 4.99*, *p*<0.001), dimeter of tuberous roots (*F_7,32_=7.09*, *p*<0.001).
