# Supplementary material for: The search for yield predictors for mature field-grown plants from juvenile pot-grown cassava (Manihot esculenta Crantz)
Source: PLoS One. 2020 May 6;15(5):e0232595. doi: 10.1371/journal.pone.0232595 (PMC7202627; doi:10.1371/journal.pone.0232595)
Supplement: S3 Fig — A: shoot fresh weight (2017 & 2018: F7,64 = 3.33, p = 0.004); B: root fresh weight (2017: F7,32 = 2.47, p = 0.038; 2018: F7,32 = 2.51, p = 0.035), C: harvest index (2017 & 2018: F7,64 = 10.95, p<0.001); D: harvest index (2017: F7,32 = 10.00, p<0.001; 2018: F7,32 = 4.47, p =) 0.001; E: number of commercial roots (207 & 2018: F7,64 = 6.33, p <0.001); F: number of commercial roots (2017: F7,32 = 5.57, p<0.001; 2018: F7,32 = 5.30, p <0.001); G: total number of roots (207 & 2018: F7,64 = 6.10, p <0.001); H: total number of roots (2017: F7,32 = 7.28, p<0.001; 2018: F7,32 = 2.52, p = 0.035). (DOCX) [file pone.0232595.s003.docx]

**
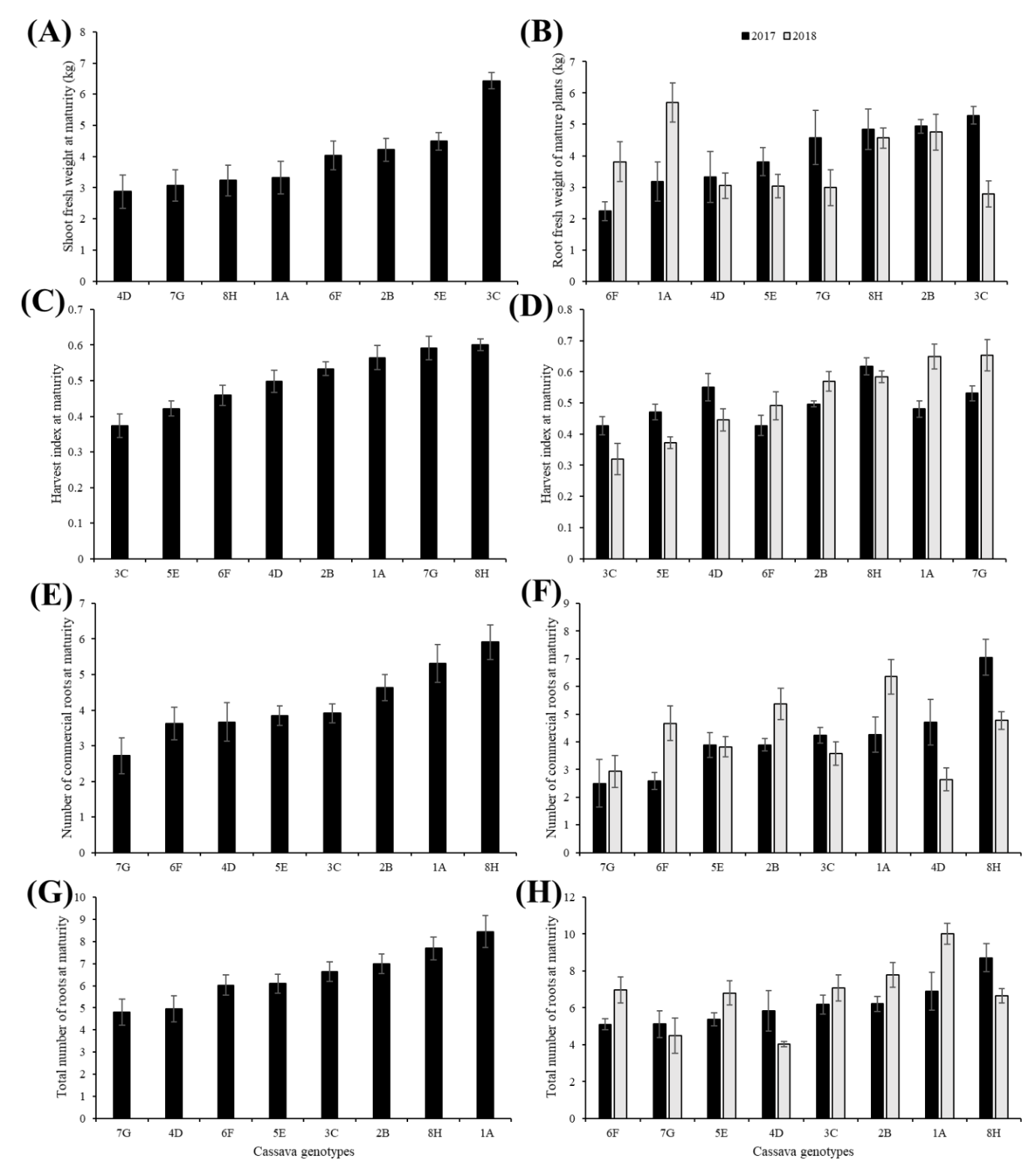
**

**Supplementary Figure S3:** Genotypic variation cassava plants grown in a field for 12-months in 2017 and 2018. **A**: shoot fresh weight (2017 & 2018: *F_7,64_=* *3.33*, *p* = 0.004); **B**: root fresh weight (2017: *F_7,32_=* *2.47*, *p* = 0.038; 2018: *F_7,32_= 2.51*, *p=* *0.035*), **C**: harvest index (2017 & 2018: *F_7,64_=* *10.95*, *p*<0.001); **D**: harvest index (2017: *F_7,32_=* *10.00*, *p*<0.001; 2018: *F_7,32_=* *4.47*, *p=*) 0.001; **E**: number of commercial roots (207 & 2018: *F_7,64_=* *6.33*, *p* <0.001); **F**: number of commercial roots (2017: *F_7,32_= 5.57*, *p*<0.001; 2018: *F_7,32_=* *5.30*, *p* *<0.001*); **G**: total number of roots (207 & 2018: *F_7,64_=* *6.10*, *p* <0.001); **H**: total number of roots (2017: *F_7,32_= 7.28*, *p*<0.001; 2018: *F_7,32_= 2.52*, *p=* *0.035*).
