# Supplementary material for: The search for yield predictors for mature field-grown plants from juvenile pot-grown cassava (Manihot esculenta Crantz)
Source: PLoS One. 2020 May 6;15(5):e0232595. doi: 10.1371/journal.pone.0232595 (PMC7202627; doi:10.1371/journal.pone.0232595)
Supplement: S1 Table — The final column shows the ANOVA between traits measured at both 30 and 45 DAP. (DOCX) [file pone.0232595.s004.docx]

**Supplementary Table S1:** Summary of descriptive statistics and summary of ANOVA results (p-value) of each shoot or root trait by genotype (8 genotypes) from juvenile cassava plants at 30 or 45 days after planting (DAP). The final column shows the ANOVA between traits measured at both 30 and 45 DAP.

| **Measure** | **Abbreviation** | **ANOVA – 30 DAP** | **Descriptive Statistics: 30 DAP** | | | **ANOVA -45 DAP** | **Descriptive Statistics: 45 DAP** | | | **ANOVA -**  **DAP** |
| --- | --- | --- | --- | --- | --- | --- | --- | --- | --- | --- |
|  |  |  | **Min.** | **Median** | **Max.** |  | **Min.** | **Median** | **Max.** |  |
| Branching density of basal roots (roots cm^-1^) | BDBR | 0.036 | 0.533 | 1.267 | 1.933 | 0.007 | 0 | 1.2 | 1.733 | 0.002 |
| Branching density of lower nodal roots (roots cm^-1^) | BDLNR | 0.424 | 0 | 1.533 | 1.933 | 0.004 | 0.667 | 1.1 | 1.467 | <0.001 |
| Branching density of upper nodal roots (roots cm^-1^) | BDUNR | 0.001 | 0.8 | 1.342 | 1.941 | <0.001 | 0 | 1.067 | 1.667 | <0.001 |
| Diameter of basal roots (mm) | DBR | 0.048 | 0.527 | 1.102 | 1.487 | <0.001 | 1.12 | 1.485 | 1.967 | <0.001 |
| Diameter of lower nodal roots (mm) | DLNR | 0.003 | 0.533 | 1.047 | 1.327 | 0.084 | 1.054 | 1.702 | 2.29 | <0.001 |
| Diameter of upper nodal roots (mm) | DUNR | <0.001 | 0.1 | 1.103 | 1.467 | 0.079 | 0 | 1.615 | 2.09 | <0.001 |
| Number of basal roots | NBR | <0.001 | 1 | 7 | 11 | <0.001 | 1 | 7 | 10 | 0.823 |
| Number of lower nodal roots | NLNR | <0.001 | 2 | 6 | 12 | <0.001 | 1 | 4 | 10 | 0.003 |
| Number of upper nodal roots | NUNR | <0.001 | 1 | 6 | 12 | <0.001 | 0 | 4 | 8 | 0.001 |
| Number of total nodal roots | NNR | <0.001 | 2 | 12 | 22 | <0.001 | 1 | 9 | 18 | 0.003 |
| Root fresh weight (mg) | RFW | 0.012 | 5 | 13.91 | 35.34 | <0.001 | 5.6 | 20.23 | 34.72 | <0.001 |
| Root dry weight (mg) | RDW | 0.068 | 0.04 | 0.525 | 1.94 | <0.001 | 0.3 | 1.8 | 5.82 | <0.001 |
| Root-to-shoot-ratio | R.S | 0.619 | 0.0439 | 0.182 | 1.143 | 0.375 | 0.0703 | 0.270 | 8.2 | 0.150 |
| Shoot fresh weight (mg) | SFW | <0.001 | 2.26 | 19.70 | 46.25 | 0.044 | 1 | 33.28 | 61.4 | <0.001 |
| Shoot dry weight (mg) | SDW | 0.002 | 0.07 | 2.575 | 7.97 | 0.036 | 0.05 | 6.025 | 10.47 | <0.001 |
| Specific root length (mg cm^-1^) | SRL | 0.112 | 193 | 1447 | 10223 | 0.881 | 60.24 | 569.3 | 2412 | <0.001 |
| Total number of roots | TRN | <0.001 | 5 | 19 | 32 | <0.001 | 2 | 15 | 26 | 0.021 |
| Total root length (cm) | TRL | 0.008 | 65.86 | 845.3 | 1636 | 0.004 | 54.22 | 1047 | 1629 | 0.007 |
