# Supplementary material for: The search for yield predictors for mature field-grown plants from juvenile pot-grown cassava (Manihot esculenta Crantz)
Source: PLoS One. 2020 May 6;15(5):e0232595. doi: 10.1371/journal.pone.0232595 (PMC7202627; doi:10.1371/journal.pone.0232595)
Supplement: S2 Table — (DOCX) [file pone.0232595.s005.docx]

**Supplementary Table S2:** Summary of descriptive statistics of the shoot and root traits data and summary of ANOVA (p-value) between field-grown cassava genotypes at 7 months after planting.

| **Measure (Unit)** | **Abbreviation** | **Descriptive statistics** | | | | **ANOVA *P*-Values** | | |
| --- | --- | --- | --- | --- | --- | --- | --- | --- |
|  |  | **Mean** | **Median** | **Minimum** | **Maximum** | **Genotype** | **Block** | **Genotype x Block** |
| Branch level number | BN | 1.58 | 1.5 | 0 | 4 | 0.002 | 0.160 | <0.001 |
| Commercial roots number | CRN | 4.21 | 4 | 0 | 12 | <0.001 | <0.001 | 0.068 |
| Feeder roots diameter (mm) | FeRD | 2.18 | 2.14 | 0 | 11.28 | 0.537 | 0.188 | 0.470 |
| Feeder roots length (cm) | FeRL | 23.62 | 24.91 | 0 | 48.5 | 0.120 | 0.102 | 0.763 |
| Feeder roots number | FeRN | 11.07 | 7 | 0 | 45 | <0.001 | 0.183 | 0.029 |
| Fibrous roots diameter (mm) | FiRD | 2.86 | 2.84 | 0 | 5.7 | 0.095 | 0.730 | 0.271 |
| Fibrous roots length (cm) | FiRL | 16.18 | 15 | 0 | 33.6 | 0.031 | 0.478 | 0.013 |
| Harvest index | HI | 0.60 | 0.60 | 0.37 | 0.82 | <0.001 | 0.410 | 0.391 |
| Leafless Stem height(cm) | LSH | 154.8 | 155 | 53.5 | 250 | 0.027 | 0.896 | 0.006 |
| Peduncle diameter (mm) | PD | 6.5 | 6.00 | 0 | 24.23 | <0.001 | <0.011 | 0.627 |
| Peduncle extent | PE | 3.56 | 5 | 0 | 5 | <0.001 | 0.233 | 0.567 |
| Peduncle length (cm) | PL | 4.2 | 4.57 | 0 | 11.67 | <0.001 | 0.190 | 0.428 |
| Primary stem diameter (mm) | PSD | 18.24 | 18.00 | 0 | 34.9 | <0.001 | <0.001 | <0.001 |
| Primary stem length (cm) | PSL | 98.61 | 103 | 20 | 184 | <0.001 | 0.935 | 0.095 |
| Primary stem number | PSN | 1.42 | 1 | 1 | 3 | <0.001 | 0.257 | 0.362 |
| Root fresh weight (kg) | fRFW | 2.80 | 2.80 | 0.4 | 5.5 | 0.003 | 0.929 | 0.152 |
| Secondary stem diameter (mm) | SSD | 11.09 | 12.33 | 0 | 22.82 | 0.047 | 0.185 | 0.002 |
| Secondary stem length (cm) | SSL | 56.80 | 55.5 | 0 | 170 | <0.001 | 0.643 | 0.003 |
| Shoot fresh weight (kg) | fSFW | 2.00 | 1.6 | 0.2 | 6.1 | <0.001 | 0.926 | 0.072 |
| Tuberous roots diameter (mm) | TRD | 50.61 | 47.88 | 29 | 83 | <0.001 | 0.003 | 0.005 |
| Tuberous roots length | TRL | 31.56 | 31.06 | 15.25 | 53.67 | 0.093 | 0.012 | 0.209 |
| Tuberous roots number | TRN | 6.16 | 5 | 2 | 15 | <0.001 | 0.017 | 0.039 |
