# Supplementary material for: The search for yield predictors for mature field-grown plants from juvenile pot-grown cassava (Manihot esculenta Crantz)
Source: PLoS One. 2020 May 6;15(5):e0232595. doi: 10.1371/journal.pone.0232595 (PMC7202627; doi:10.1371/journal.pone.0232595)
Supplement: S3 Table — Loading scores of traits on each component and the proportion of variation explained with the first seven significant components are presented. Components with eigenvalues >1 are considered significant. Bold indicates variable loading scores with the greatest loads on each component. Genotype means data (n = 3) were used for PCA. (DOCX) [file pone.0232595.s006.docx]

**Supplementary Table S3: Results of principal component analysis (PCA) on 22 traits, (8 shoot and 14 root traits) of 8 cassava genotypes grown in the field for 7 months. Loading scores of traits on each component and the proportion of variation explained with the first seven significant components are presented.** Components with eigenvalues >1 are considered significant. **B**old indicates variable loading scores with the greatest loads on each component. Genotype means data (n = 3) were used for PCA.

| **Trait (unit)** | **Abbreviation** | **Principal Components** | | | | | | |
| --- | --- | --- | --- | --- | --- | --- | --- | --- |
|  |  | PC1 | PC2 | PC3 | PC4 | PC5 | PC6 | PC7 |
| Primary stem number | PSN | 0.07 | 0.47 | 0.18 | **-0.51** | 0.05 | -0.16 | -0.46 |
| Branch level number | BN | **0.71** | -0.28 | -0.39 | 0.00 | 0.21 | 0.05 | 0.02 |
| Primary stem diameter (mm) | PSD | **0.73** | 0.04 | -0.18 | 0.29 | 0.05 | -0.10 | 0.11 |
| Primary stem length (cm) | PSL | -0.19 | **0.59** | 0.55 | 0.25 | 0.28 | 0.01 | 0.19 |
| Secondary stem length (cm) | SSL | **0.76** | -0.49 | -0.25 | -0.12 | 0.07 | 0.09 | -0.04 |
| Secondary stem diameter (mm) | SSD | **0.74** | -0.39 | -0.32 | -0.07 | 0.06 | -0.04 | -0.18 |
| Leafless Stem height(cm) | LSH | **0.58** | 0.19 | 0.42 | 0.18 | 0.41 | 0.11 | 0.17 |
| Shoot fresh weight (kg) | fSFW | **0.76** | 0.02 | 0.41 | -0.10 | -0.32 | -0.22 | 0.06 |
| Root fresh weight (kg) | fRFW | **0.75** | 0.31 | 0.38 | -0.10 | 0.04 | -0.14 | -0.13 |
| Harvest index | HI | -0.39 | 0.39 | -0.25 | 0.17 | **0.64** | 0.05 | -0.05 |
| Peduncle extent | PE | 0.03 | **0.74** | -0.30 | -0.15 | -0.08 | -0.31 | 0.23 |
| Peduncle length (cm) | PL | -0.20 | **0.74** | -0.08 | -0.04 | -0.13 | -0.19 | -0.08 |
| Peduncle diameter (mm) | PD | 0.06 | **0.55** | -0.54 | 0.13 | -0.28 | -0.21 | 0.22 |
| Tuberous roots number | TRN | 0.28 | **0.57** | -0.33 | -0.16 | -0.06 | 0.56 | 0.03 |
| Tuberous roots length | fTRL | 0.37 | 0.39 | 0.05 | **0.44** | 0.37 | 0.04 | -0.15 |
| Tuberous roots diameter (mm) | TRD | **0.55** | 0.17 | 0.01 | 0.32 | -0.09 | -0.17 | -0.09 |
| Fibrous roots length (cm) | FiRL | 0.03 | 0.28 | 0.06 | 0.41 | **-0.61** | 0.44 | -0.03 |
| Fibrous roots diameter (mm) | FiRD | 0.02 | -0.09 | 0.31 | **0.70** | -0.24 | 0.00 | -0.38 |
| Feeder roots number | FeRN | 0.40 | 0.42 | **0.42** | -0.42 | -0.09 | -0.02 | -0.26 |
| Feeder roots length (cm) | FeRL | 0.02 | -0.10 | 0.51 | -0.28 | 0.01 | **0.61** | 0.20 |
| Feeder roots diameter (mm) | FeRD | 0.28 | -0.12 | 0.33 | -0.03 | -0.06 | -0.23 | **0.72** |
| Commercial roots number | CRN | 0.48 | **0.54** | -0.38 | -0.08 | -0.01 | 0.40 | 0.10 |
| Eigen Value | | 4.9 | 3.8 | 2.5 | 1.8 | 1.5 | 1.4 | 1.3 |
| Percentage Variation | | 22.0 | 17.4 | 11.5 | 8.1 | 6.9 | 6.5 | 5.8 |
| Cumulative Percentage Variation | | 22.0 | 39.4 | 51.0 | 59.0 | 65.9 | 72.4 | 78.1 |
